# Supplementary material for: The lipid A acylation pattern of Coxiella burnetii prevents detection and clearance by the non-canonical inflammasome in primary murine macrophages
Source: PLoS Pathog. 2026 Jul 24;22(7):e1014465. doi: 10.1371/journal.ppat.1014465 (PMC13436776; doi:10.1371/journal.ppat.1014465)

Figure 3: BMDM were stimulated with OMVs (2 µg/ml) for 8h, infected with *L. pneumophila* Δ*flaA* for 8h, infected with *C. burnetii* wt or Δ*dotA* for 32h or infected with *C. burnetii* wt or Δ*dotA* for 24h with subsequent infection with *L. pneumophila* Δ*flaA* or stimulation with OMVs for 8h under normoxia (N). Immunoblot analysis was performed using antibodies against GBP1, caspase 11, cleaved GSDMD, NLRP3, uncleaved IL1β, cleaved IL1β, and actin as loading control.

GBP1 and corresponding actin

GBP1 N

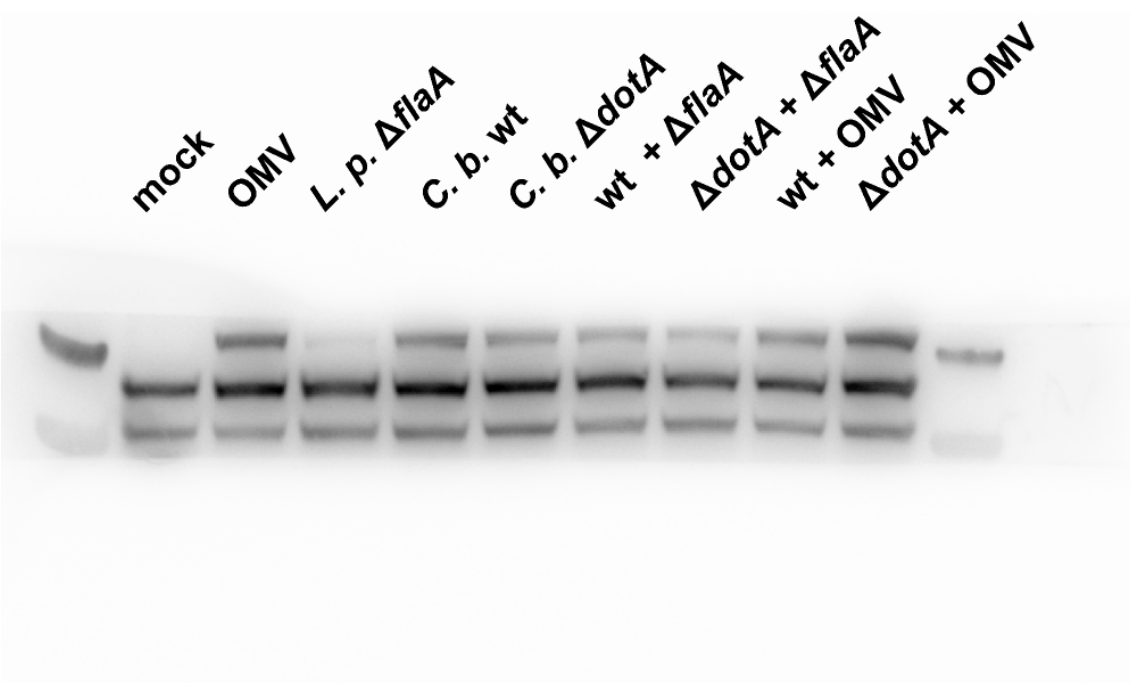

Actin N

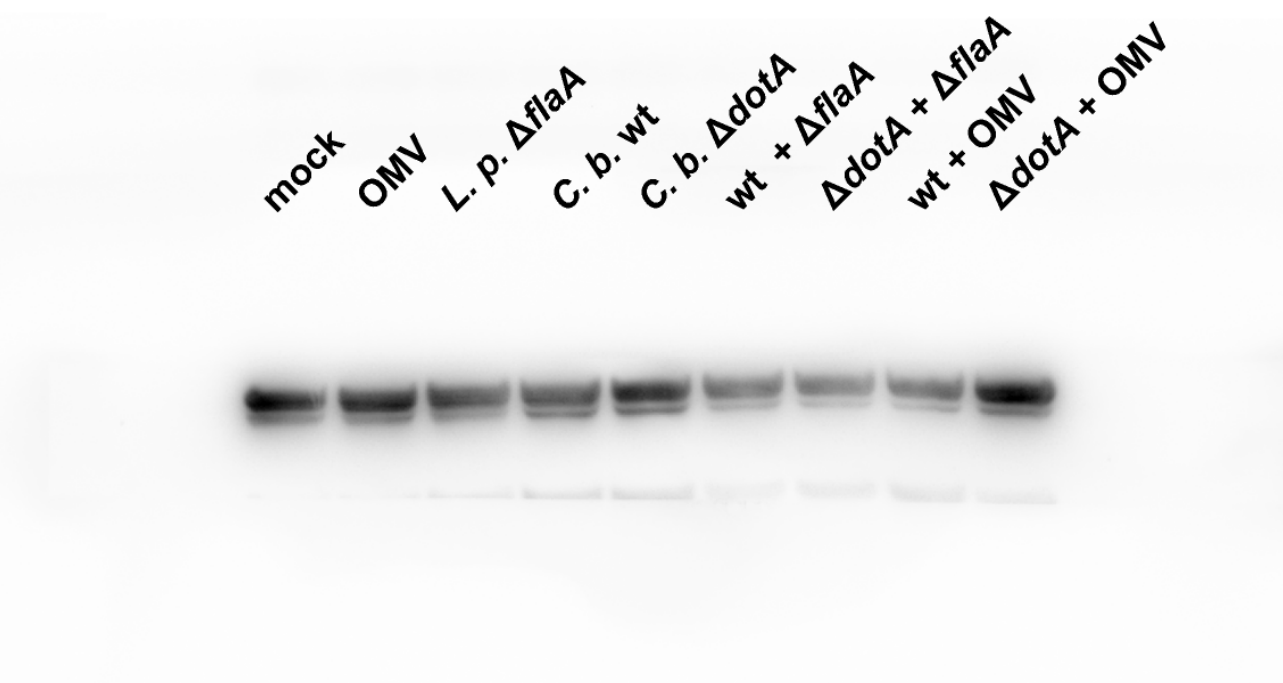

Cleaved GSDMD and corresponding actin

cleaved GSDMD N

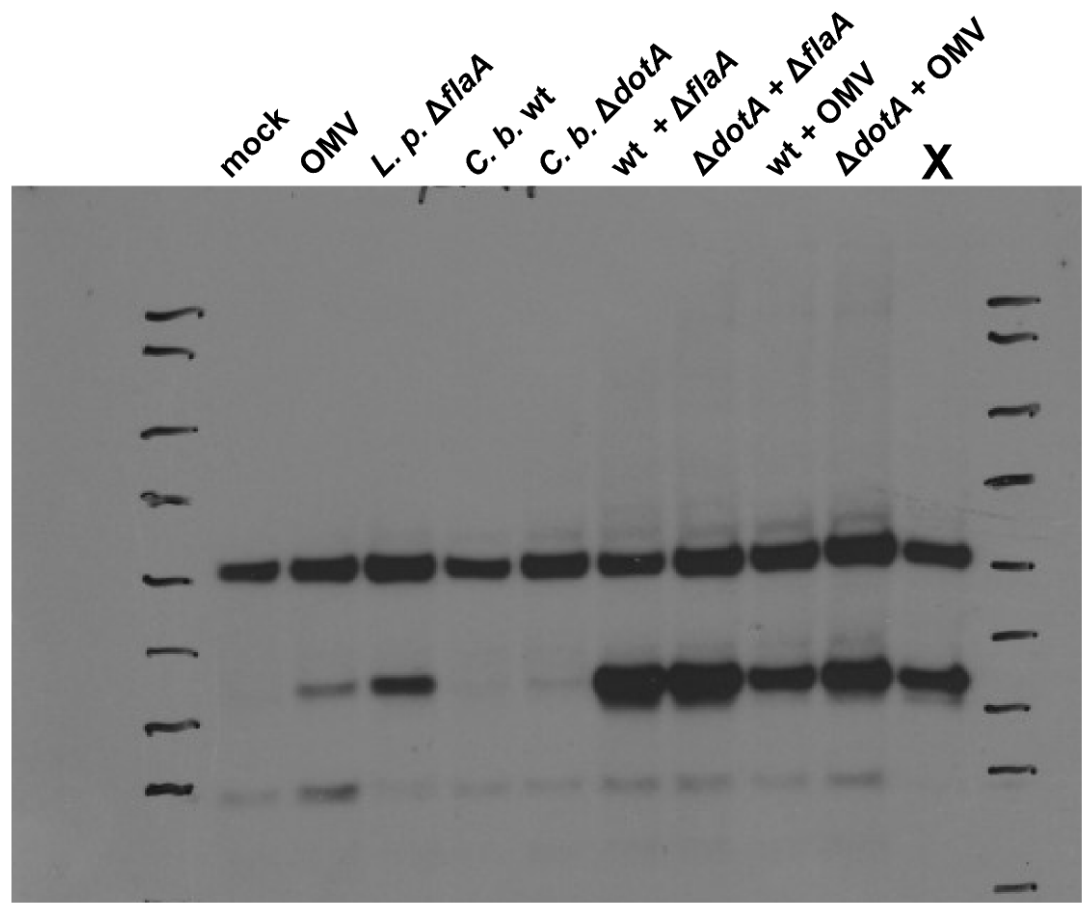

Actin N

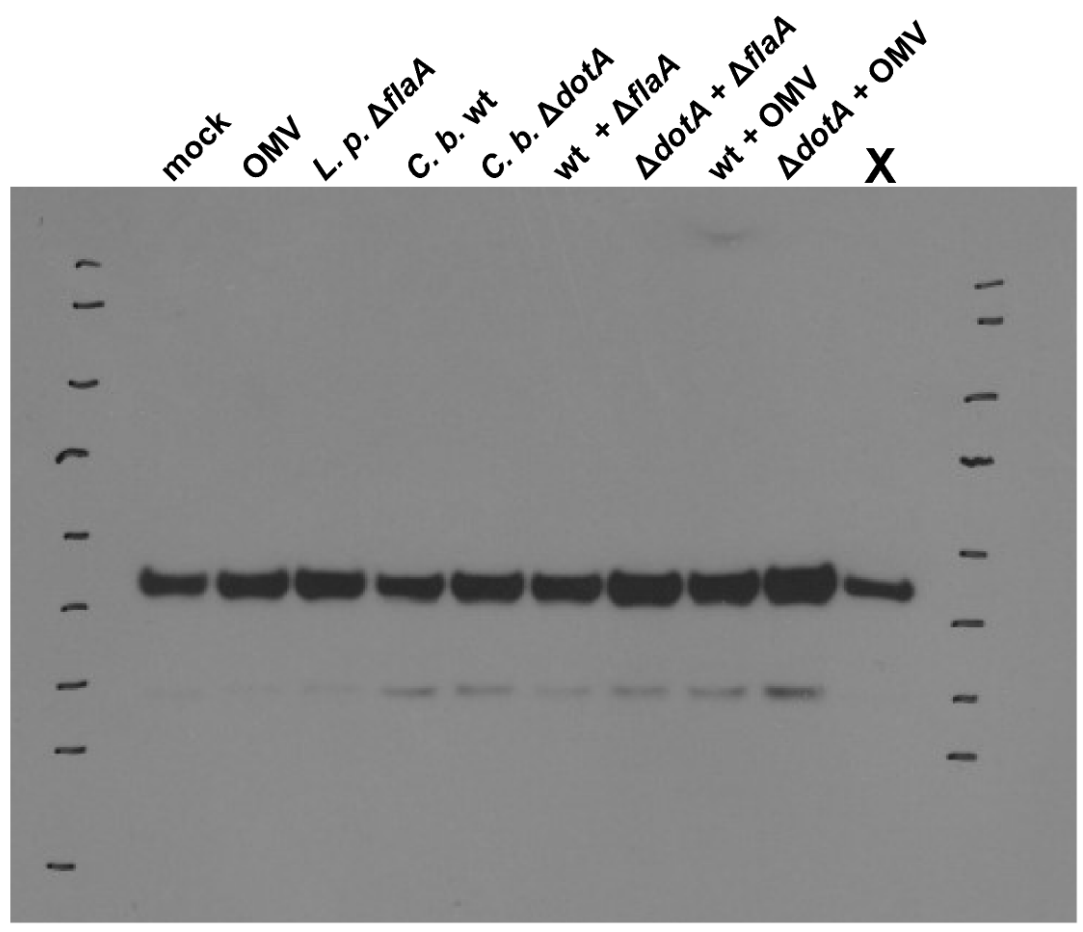

Caspase 11 and corresponding actin

Caspase-11 N

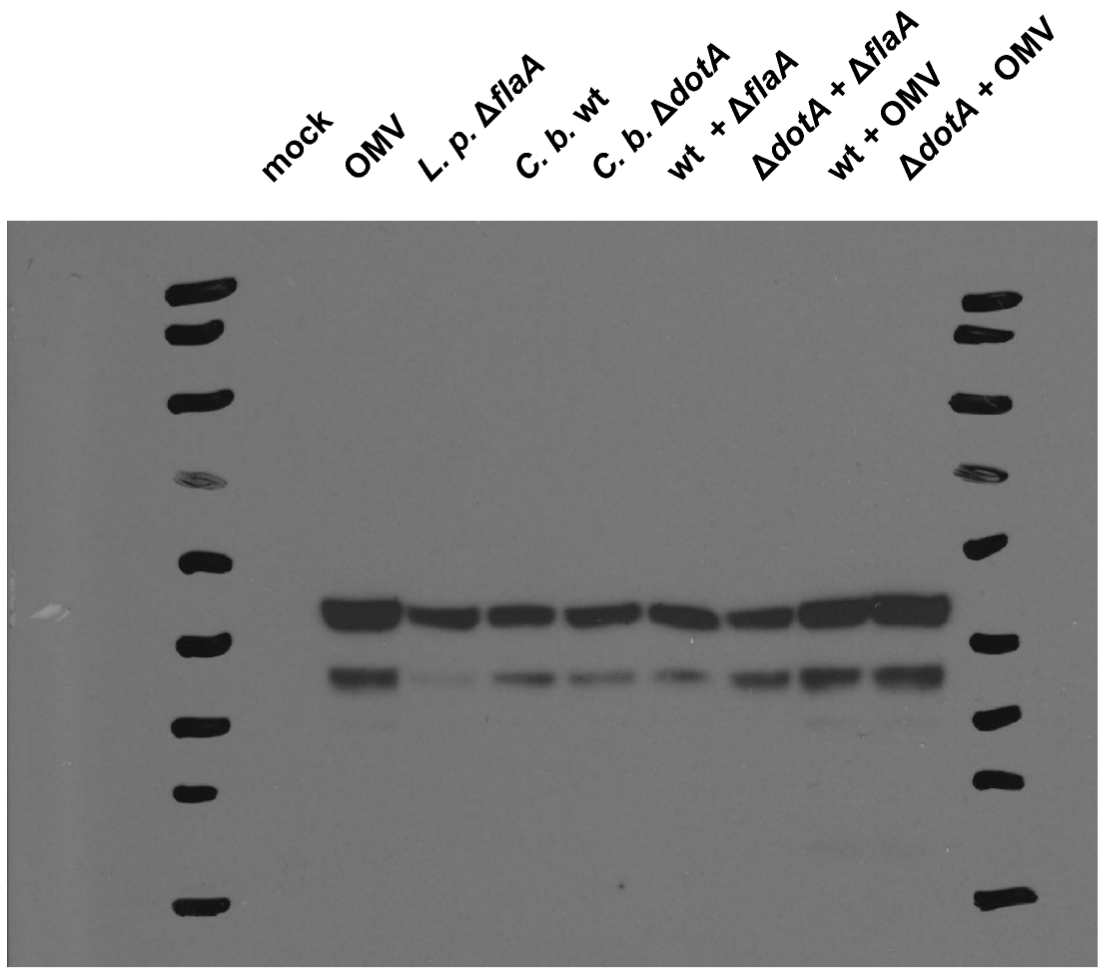

Actin N

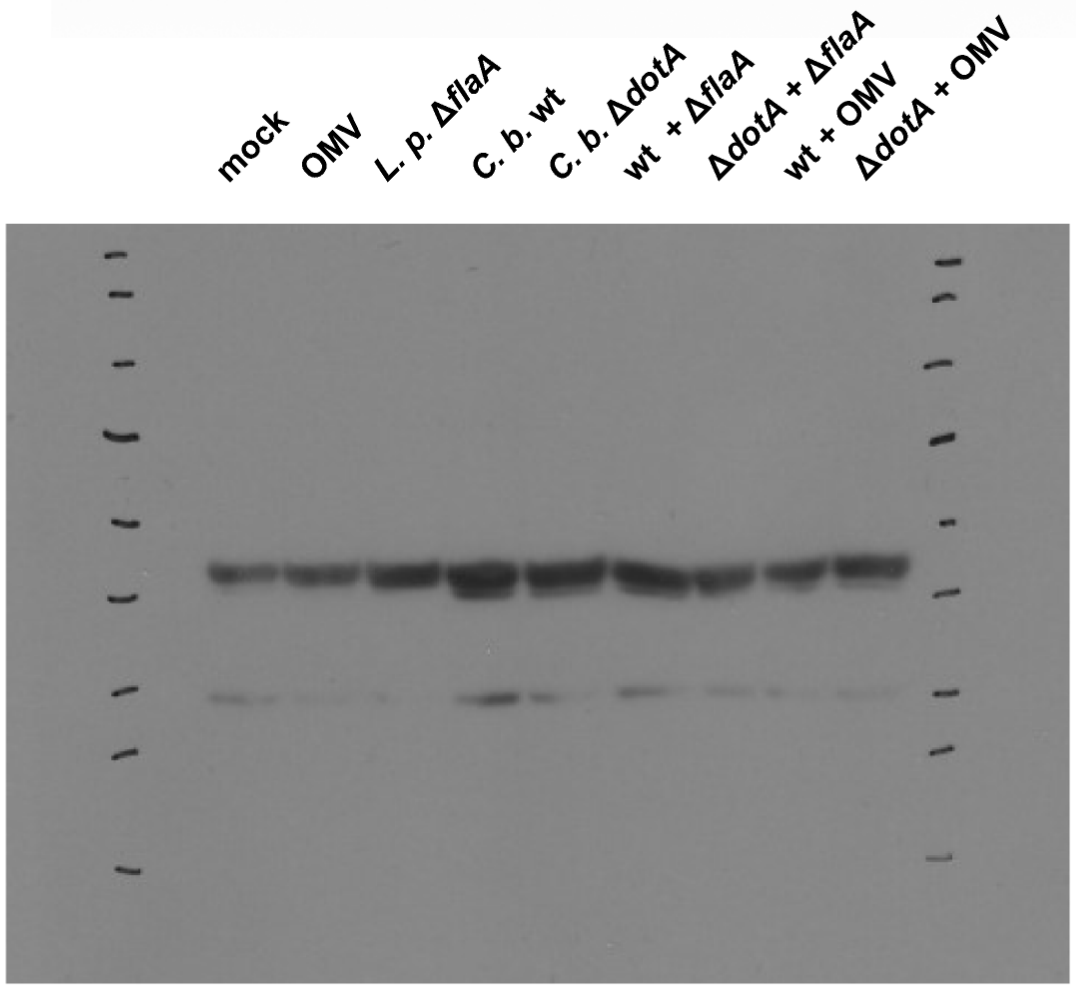

NLRP3 and corresponding actin

NLRP3 N

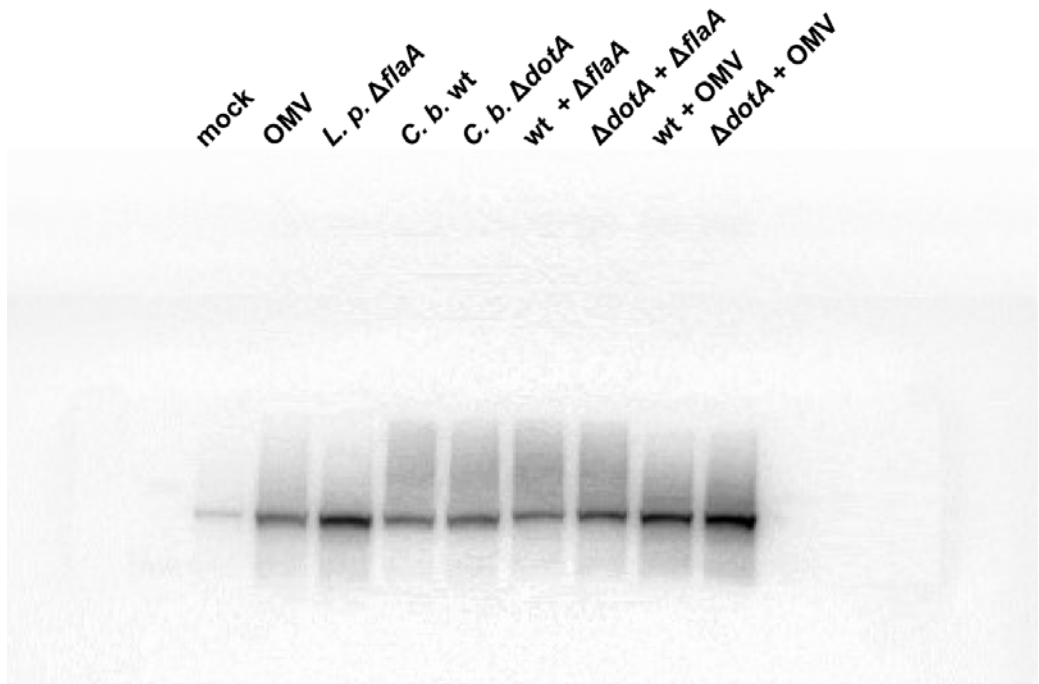

Actin N

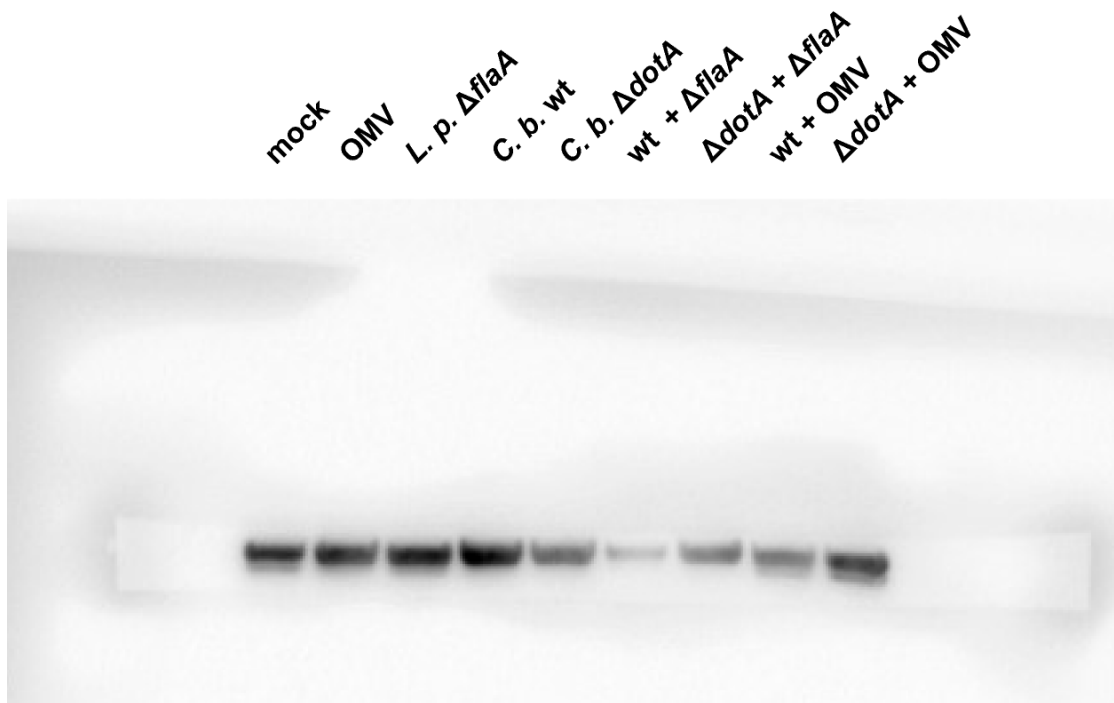

Uncleaved IL1β and corresponding actin

uncleaved IL1ß N

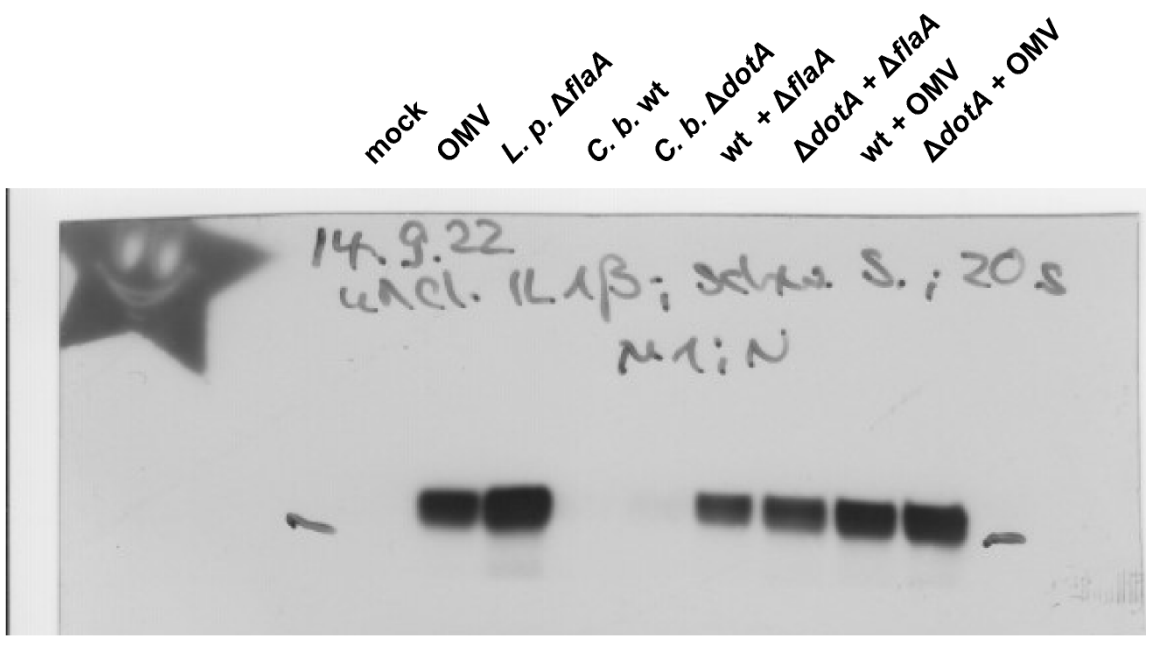

Actin N

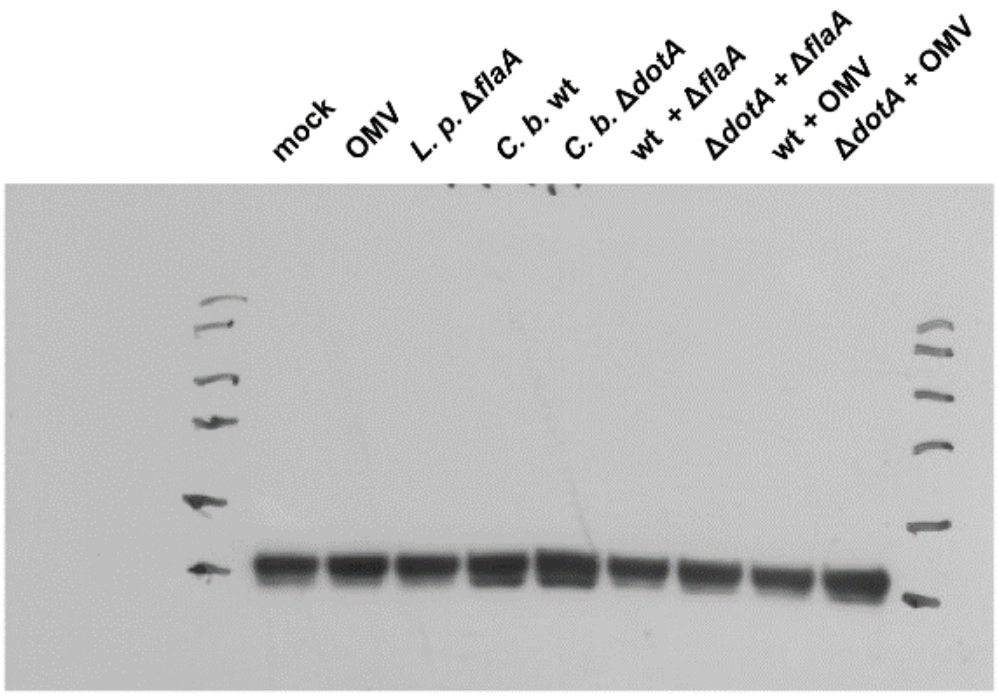

Cleaved IL1β and corresponding actin

cleaved IL1ß N

Actin N

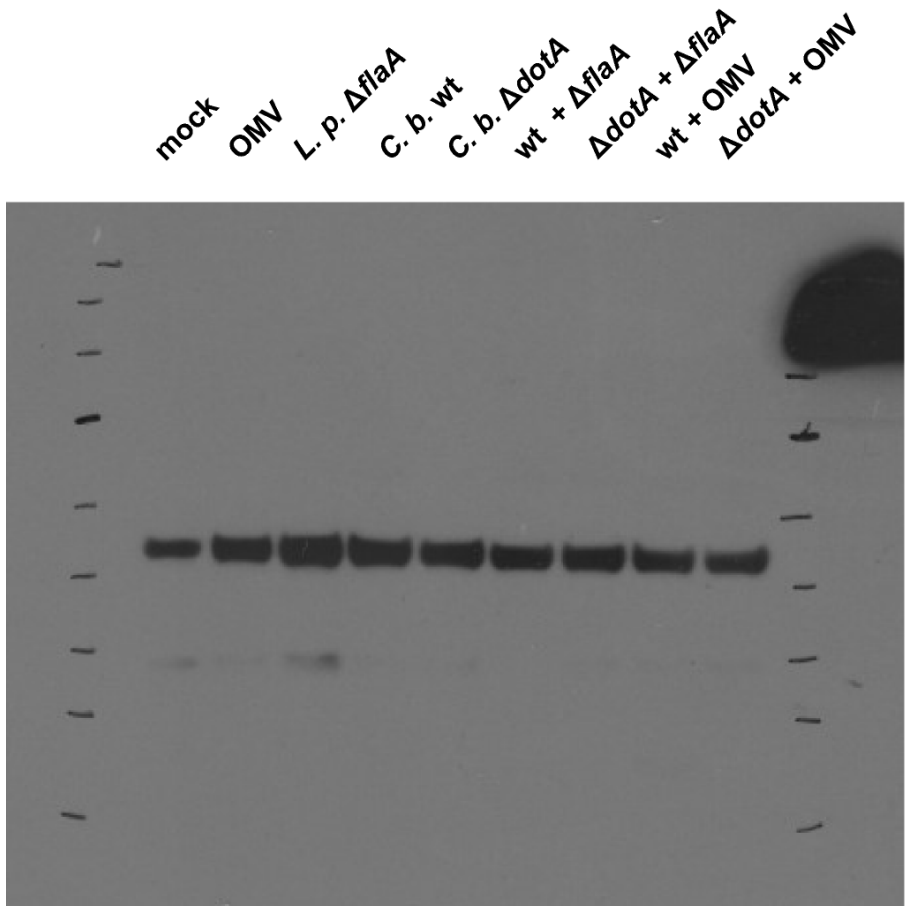

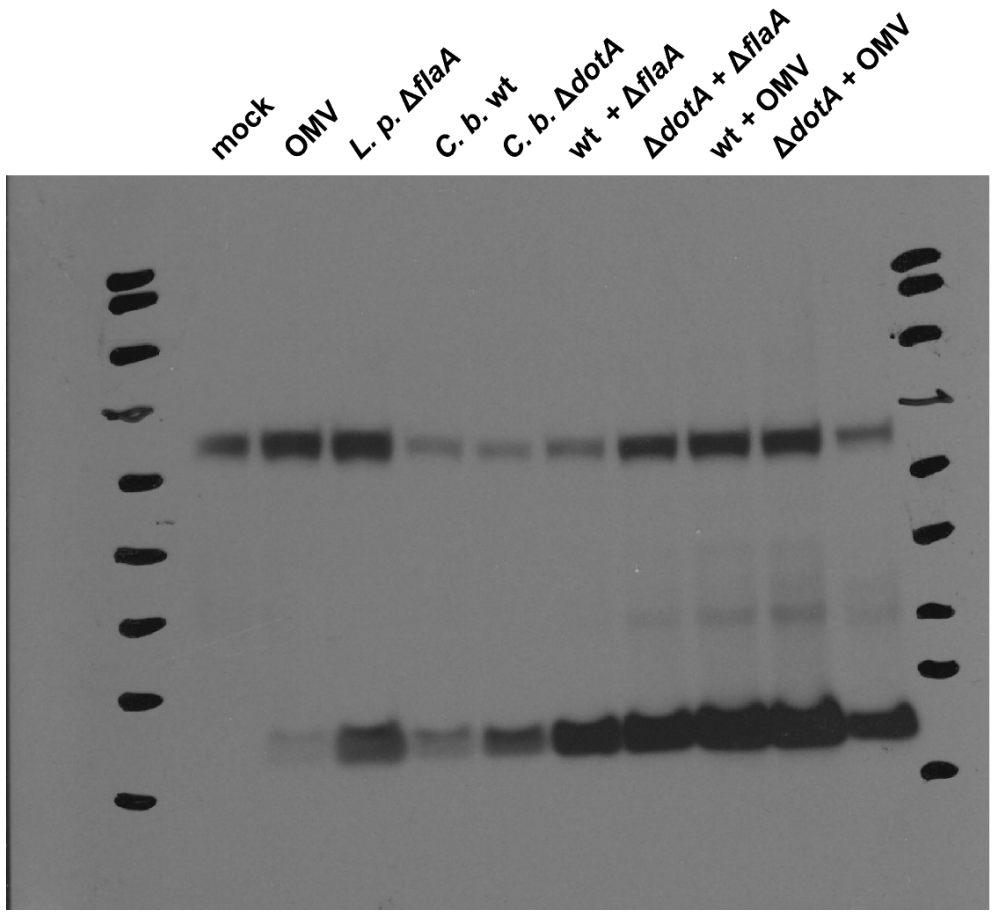

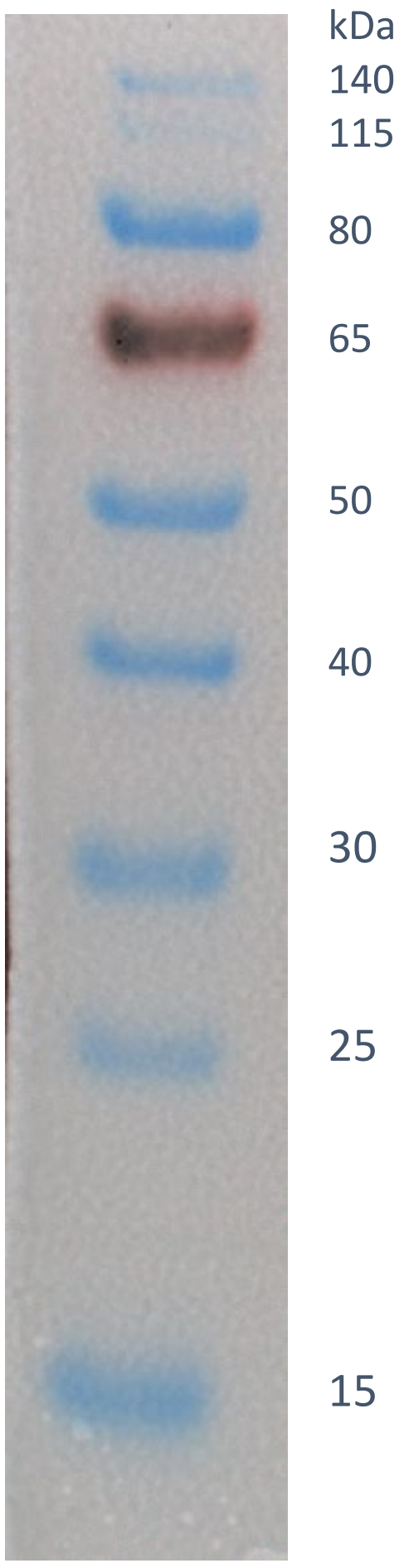

Figure 5: BMDM were stimulated with OMVs (2 µg/ml) for 8h, infected with *L. pneumophila* Δ*flaA* for 8h, infected with *C. burnetii* wt or Δ*dotA* for 32h or infected with *C. burnetii* wt or Δ*dotA* for 24h with subsequent infection with *L. pneumophila* Δ*flaA* or stimulation with OMVs for 8h under hypoxic conditions (H - 0.5% O_2). Immunoblot analysis was performed using antibodies against GBP1, caspase 11, cleaved GSDMD, NLRP3, uncleaved IL1β, cleaved IL1β, and actin as loading control. Importantly, the same membrane was used to analyze the expression of GBP1 and NLRP3. Therefore, the actin blot is the same.

GBP1 and corresponding actin

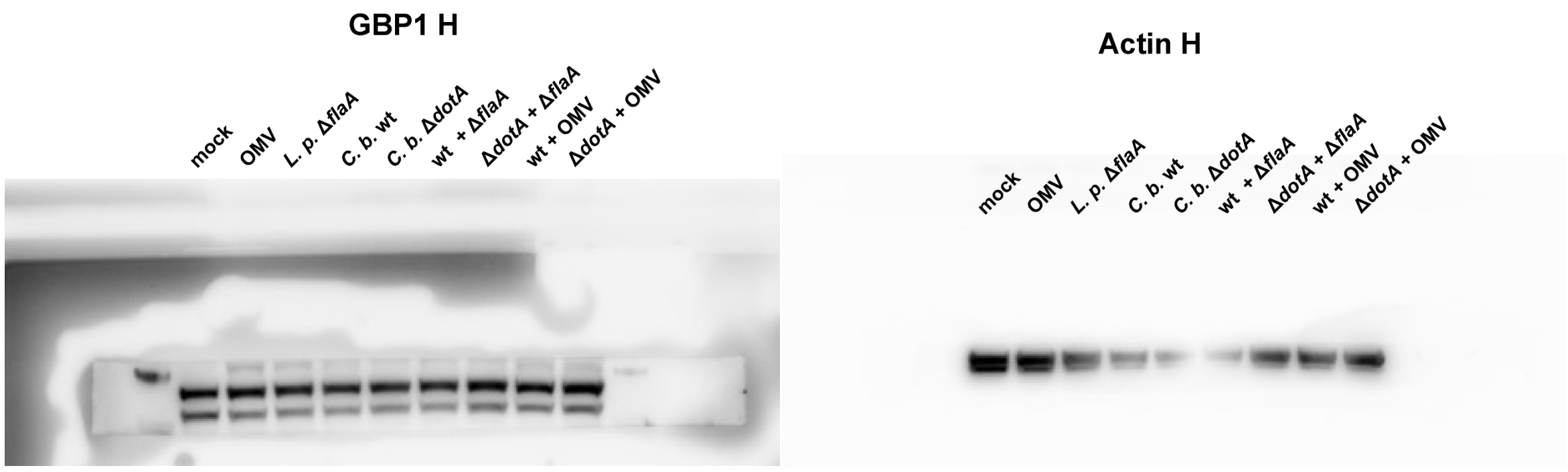

Cleaved GSDMD and corresponding actin

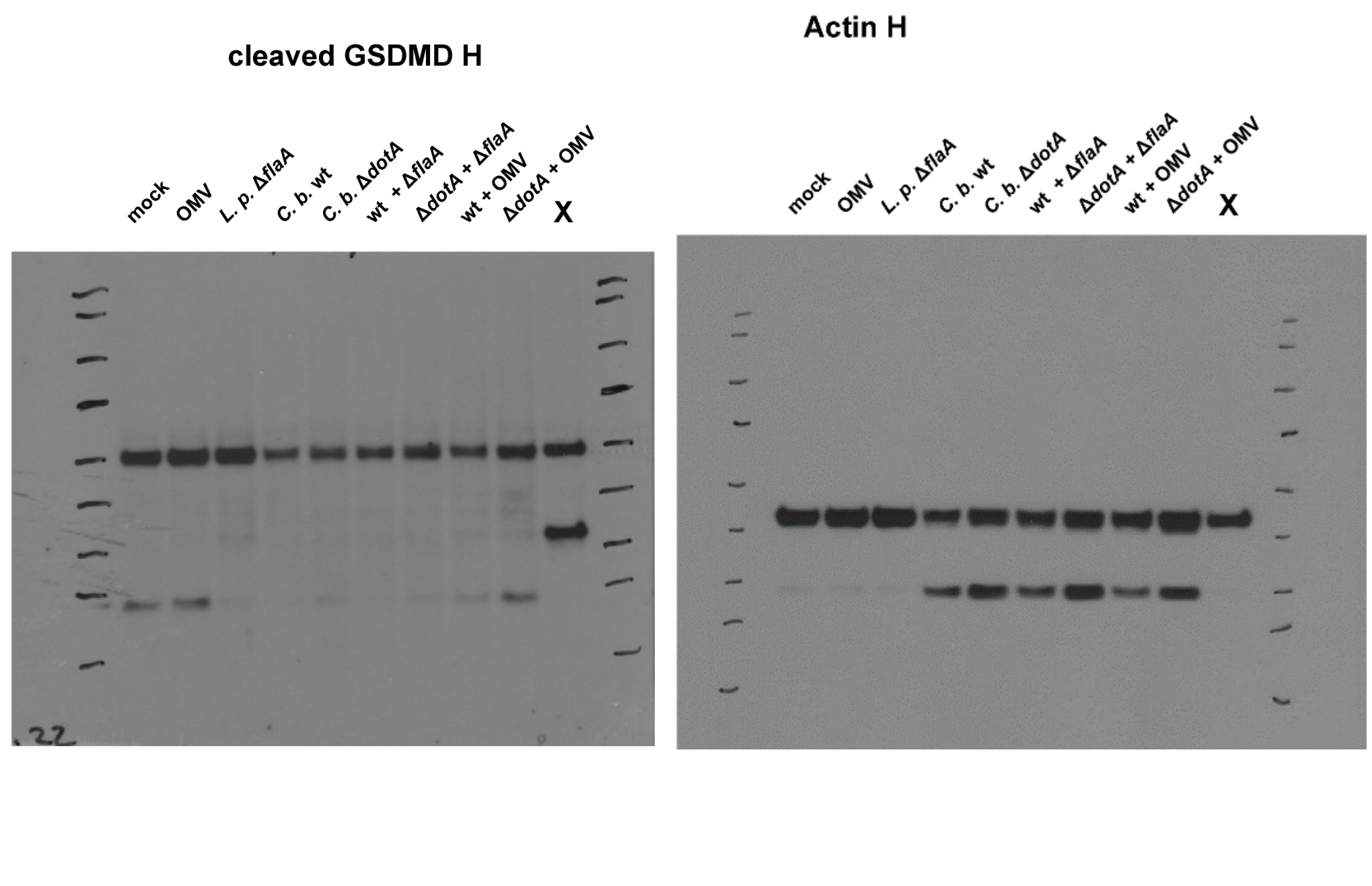

Caspase 11 and corresponding actin

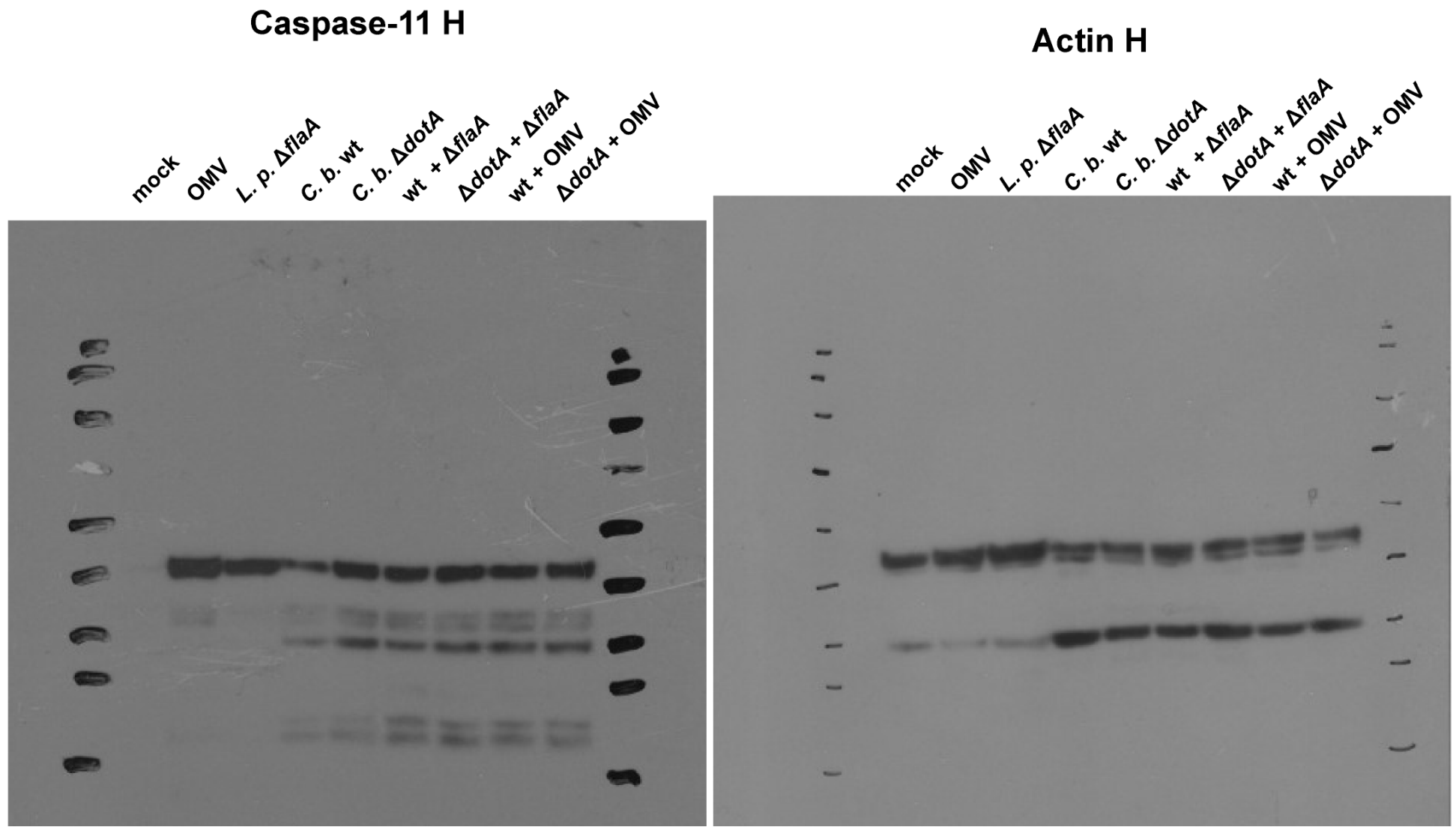

NLRP3 and corresponding actin

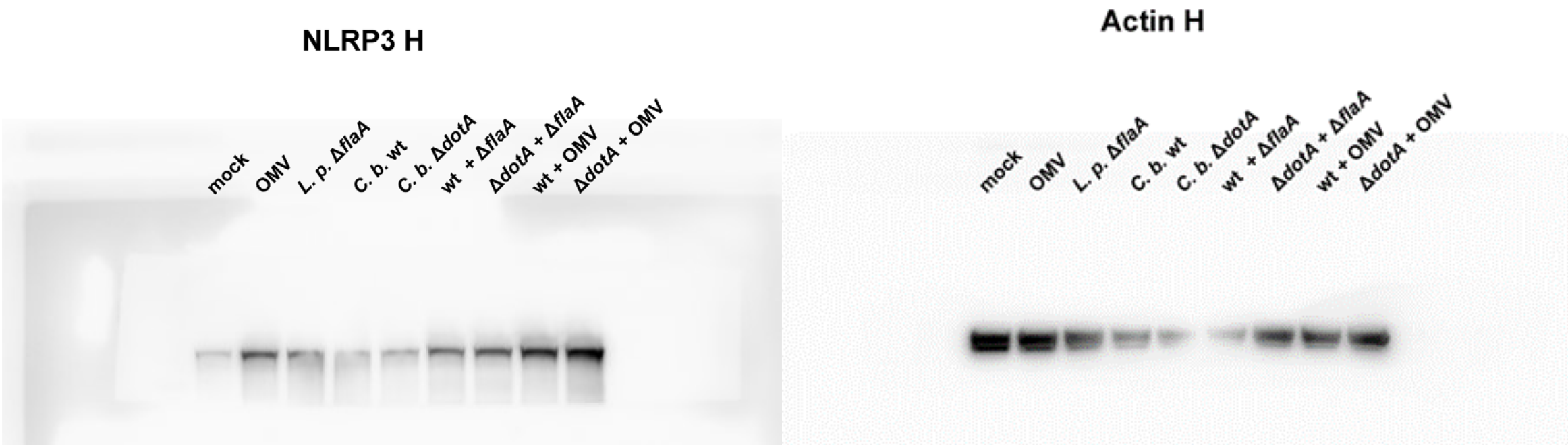

Uncleaved IL1β and corresponding actin

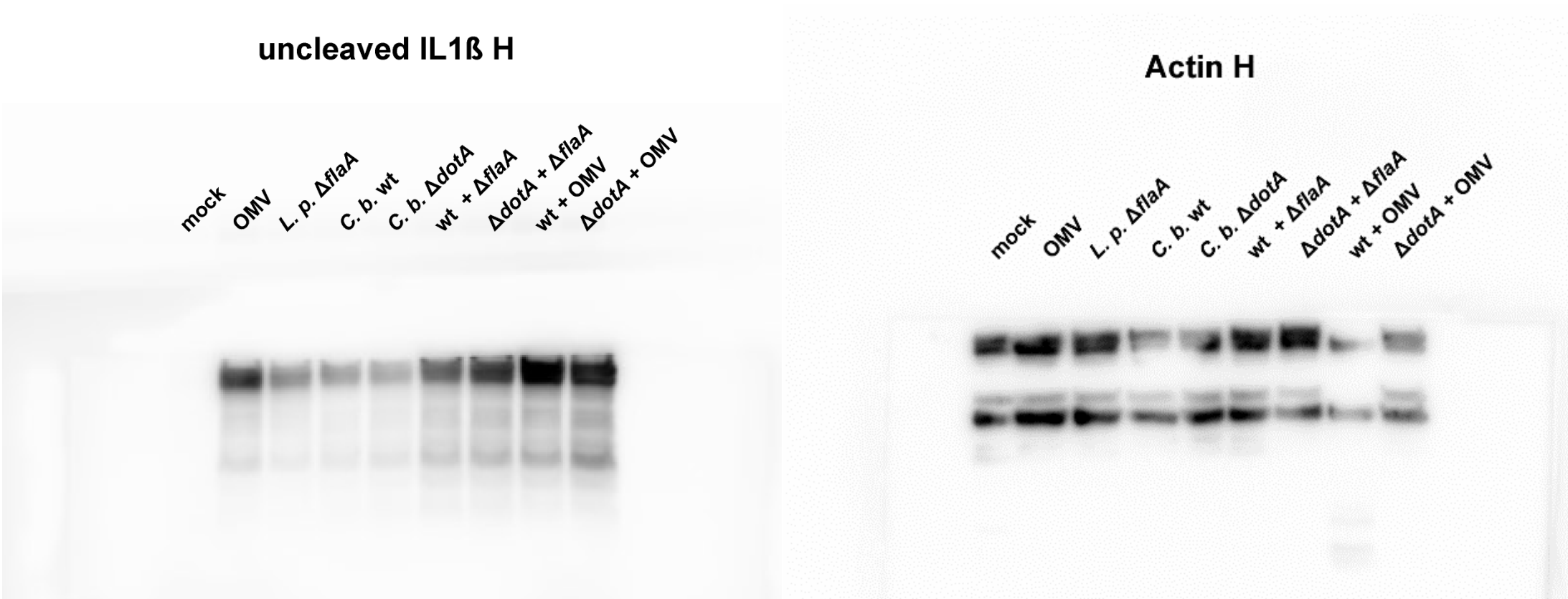

Cleaved IL1β and corresponding actin

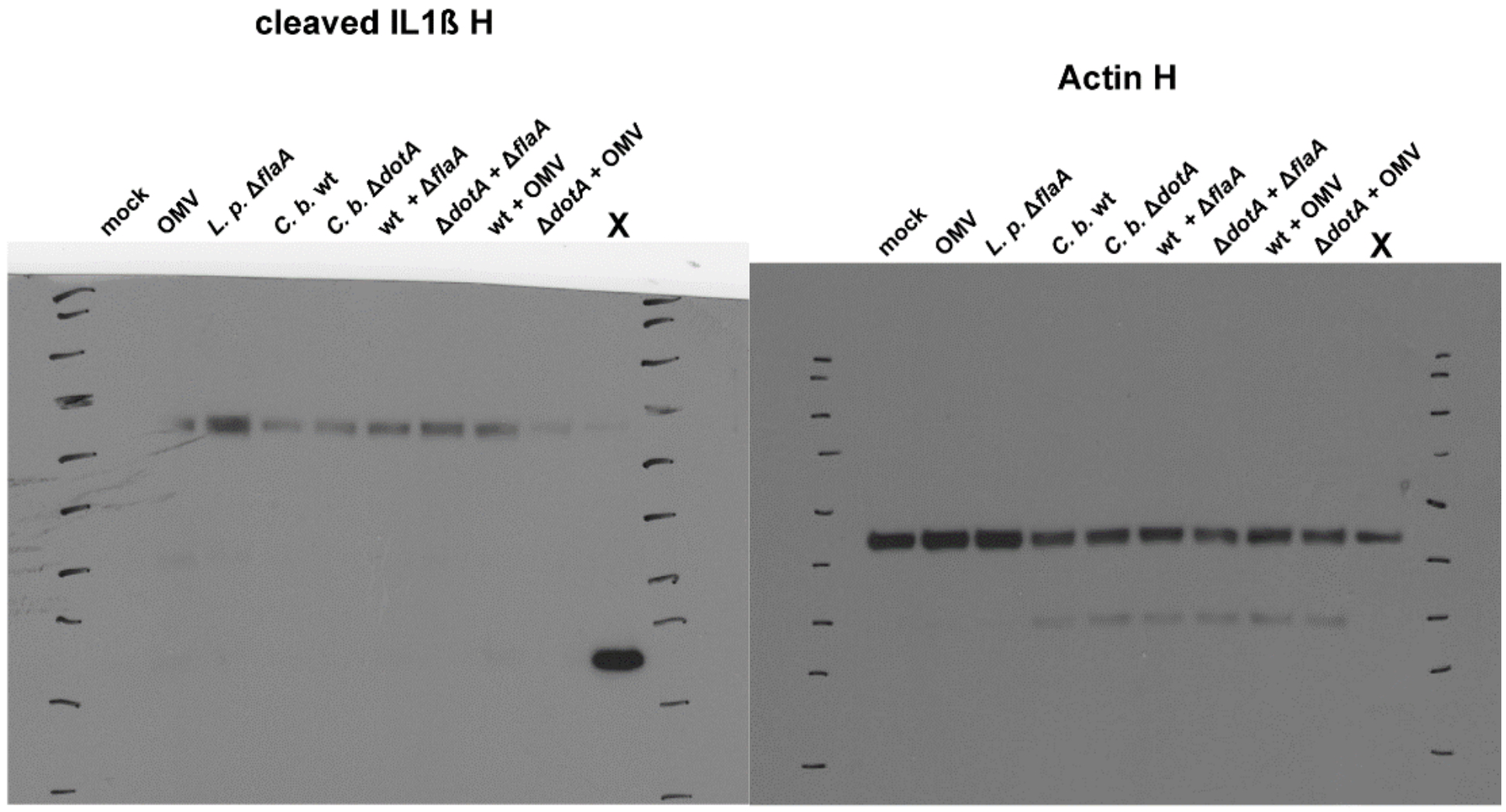

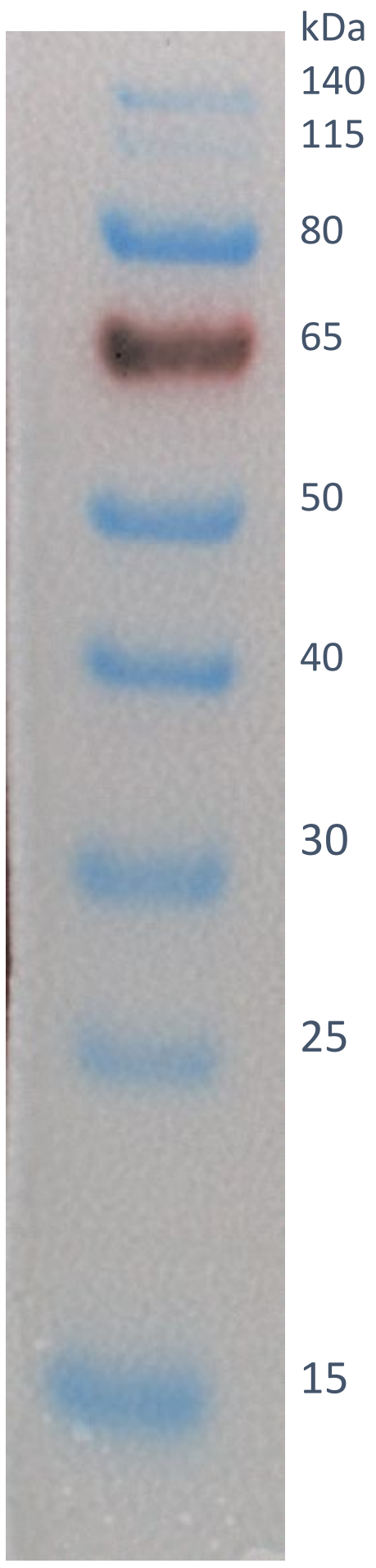

Figure 7: ACOD1$^{+/-}$ (ctrl) and ACOD1$^{-/-}$ BMDM were stimulated with OMVs, LPS + Nigericin, or infected with wt and Δ*dotA* *C. burnetii* at MOI 3 with or without additional stimulation with OMVs. Immunoblot analysis was conducted using antibodies against caspase 11, cleaved GSDMD, and actin as loading control.

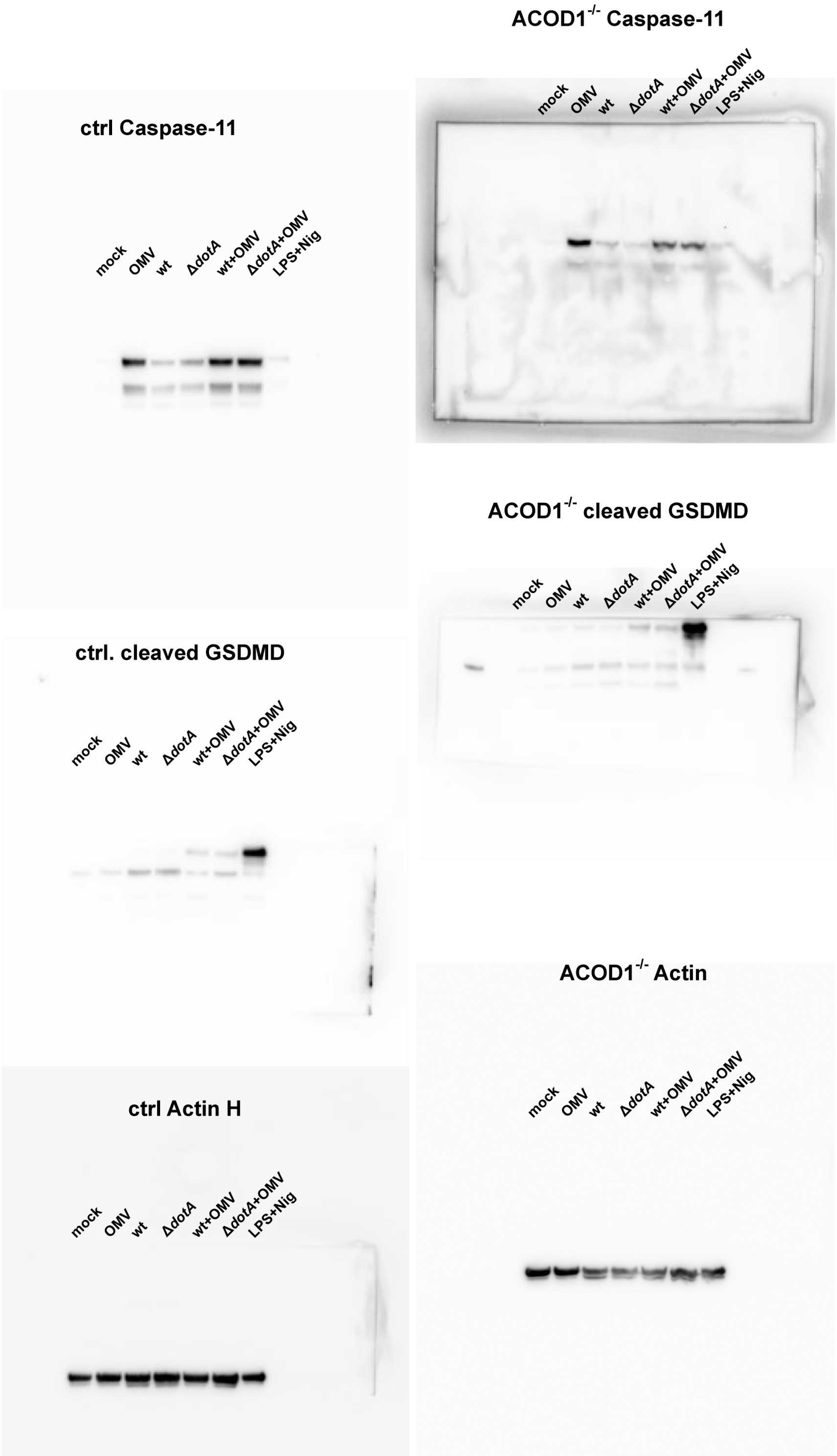

Figure 10: *C. burnetii* wild-type (WT) and the two different mutants expressing LpxA, LpxD, LpxL and LpxM (Mut1 and Mut2) were subjected to immunoblot analysis using antibodies against Flag (to detect LpxD (~39 kDa)), HA (to detect LpxA (~28 kDa) and LpxL (~36 kDa)), His (to detect LpxM (~38 kDa) and *C. burnetii*.

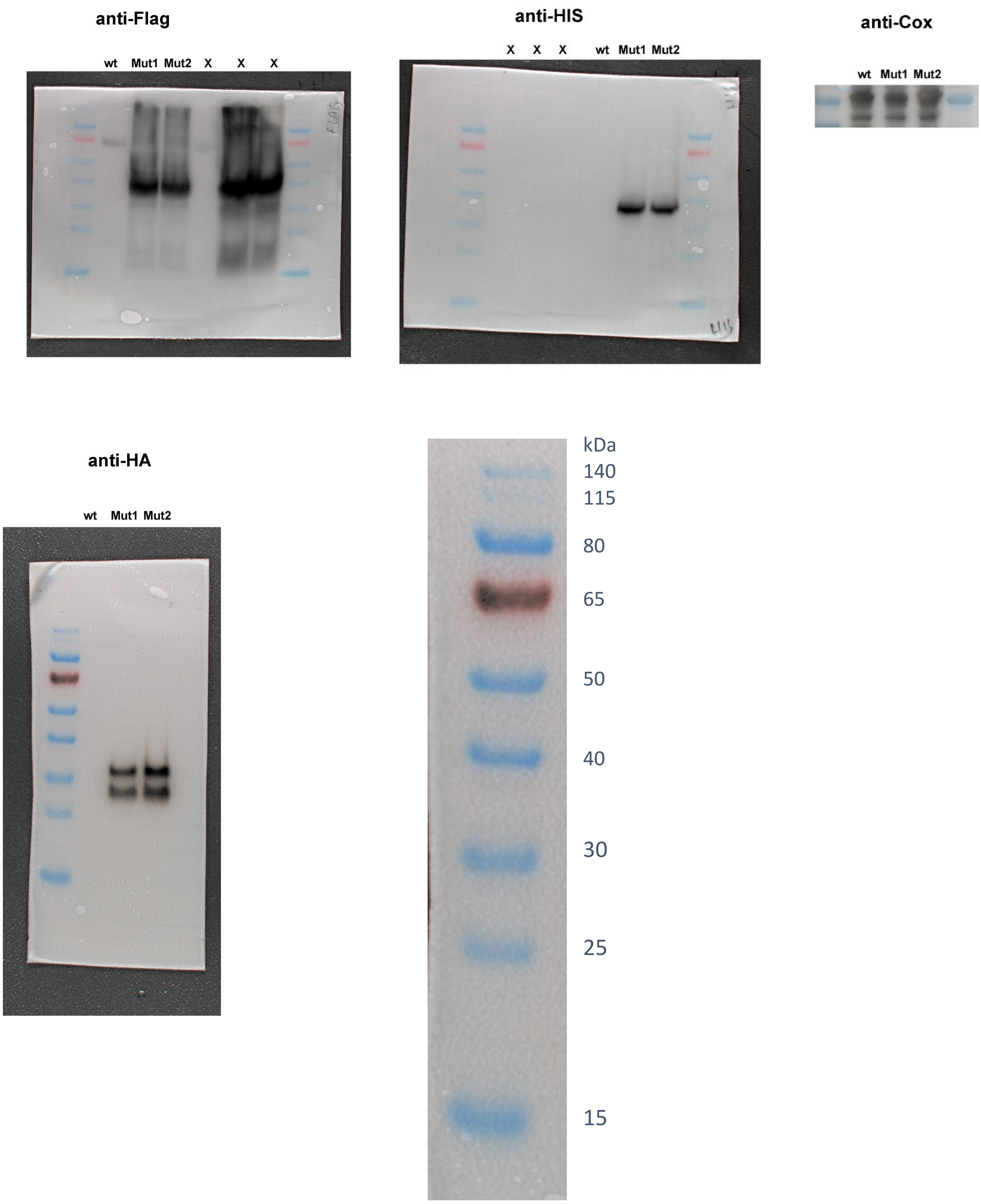

Figure S1: BMDM were either not treated or infected with *C. burnetii* wt for 32h or infected with *C. burnetii* wt for 24h with subsequent infection with *L. pneumophila* Δ*flaA* for 8h. For all infections a MOI of 3 was used. Immunoblot analysis was performed using antibodies against caspase 11 and actin as loading control.

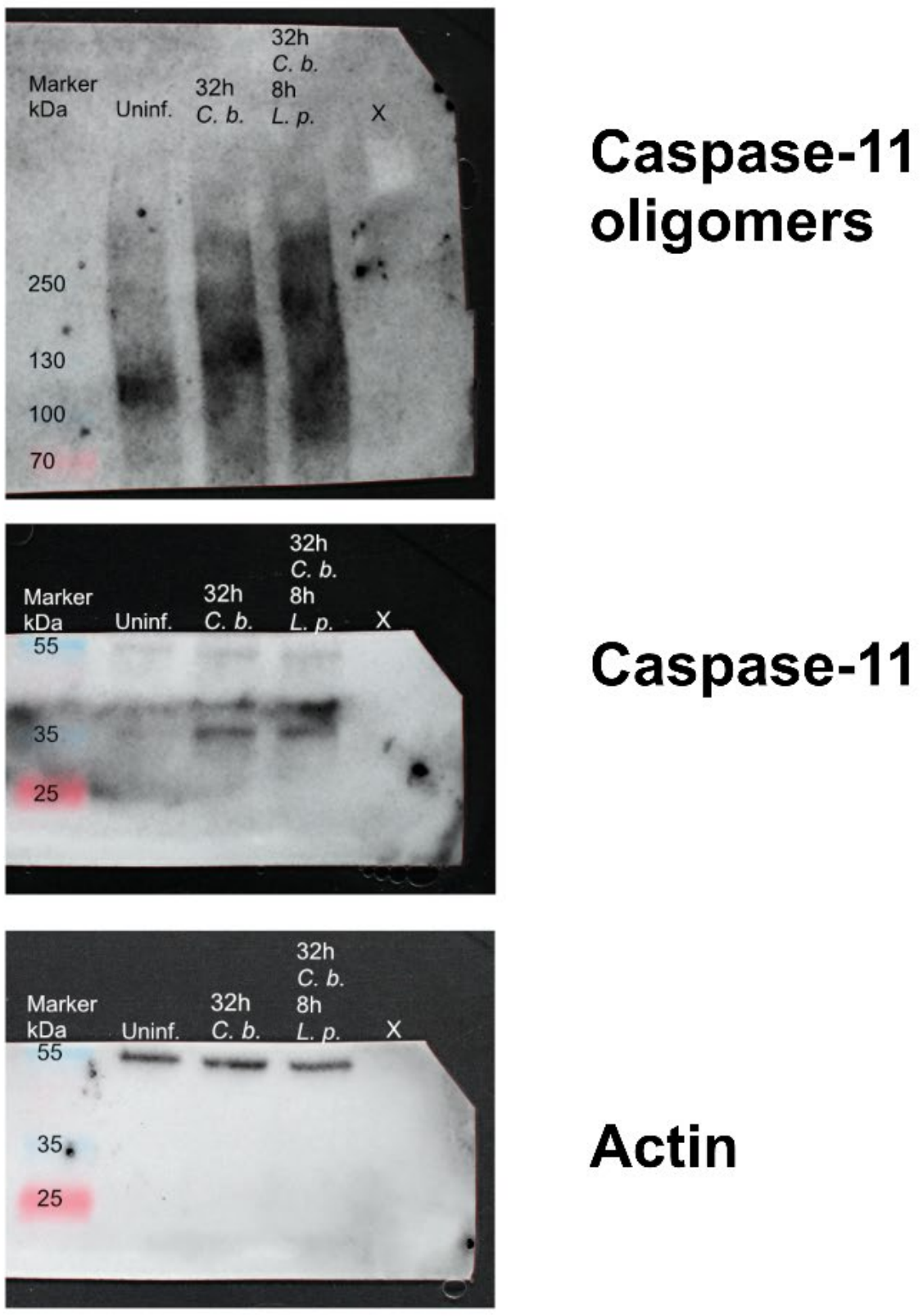

Supplement: Supplementary file 1 [file ppat.1014465.s005.pdf]
