# Supplementary material for: The lipid A acylation pattern of Coxiella burnetii prevents detection and clearance by the non-canonical inflammasome in primary murine macrophages
Source: PLoS Pathog. 2026 Jul 24;22(7):e1014465. doi: 10.1371/journal.ppat.1014465 (PMC13436776; doi:10.1371/journal.ppat.1014465)
Supplement: Supplementary file 2 [file ppat.1014465.s001.docx]

S1 Fig

**S1 Fig: Infection with *C. burnetii* fails to potently induce caspase 11 oligomerization in murine BMDM.** BMDM were either not treated or infected with *C. burnetii* wt for 32h or infected with *C. burnetii* wt for 24h with subsequent infection with *L. pneumophila* Δ*flaA* for 8h. For all infections a MOI of 3 was used. Immunoblot analysis was performed using antibodies against caspase 11 and actin as loading control. One representative blot from at least three independent experiments is shown.
