# Supplementary material for: The lipid A acylation pattern of Coxiella burnetii prevents detection and clearance by the non-canonical inflammasome in primary murine macrophages
Source: PLoS Pathog. 2026 Jul 24;22(7):e1014465. doi: 10.1371/journal.ppat.1014465 (PMC13436776; doi:10.1371/journal.ppat.1014465)
Supplement: Supplementary file 3 [file ppat.1014465.s002.docx]

S2 Fig

**S2 Fig: Plasmid map of pKM244mod-*lpxM*-His-*lpxL*-HA-*lpxD*-3xFlag-*lpxA*-HA.** This shuttle vector contains the RSF1010 *oriV* as replication origin and ampicillin and chloramphenicol resistance cassettes for selection in *Escherichia coli* or *C. burnetii*, respectively. The expression of LpxA-HA, LpxD-Flag, LpxL-HA and LpxM-His is driven by the IPTG-inducible *tac* promotor.
