# Supplementary material for: The lipid A acylation pattern of Coxiella burnetii prevents detection and clearance by the non-canonical inflammasome in primary murine macrophages
Source: PLoS Pathog. 2026 Jul 24;22(7):e1014465. doi: 10.1371/journal.ppat.1014465 (PMC13436776; doi:10.1371/journal.ppat.1014465)
Supplement: Supplementary file 5 [file ppat.1014465.s004.docx]

**SUPPORTING INFORMATION – RAW DATA**

**For figure 1 A:**

| **Strain** | **Day** | **OD_600_** |
| --- | --- | --- |
| wt | 0 | 0  0  0  0 |
| Δ*dotA* | 0 | 0  0  0  0 |
| wt | 2 | 0.019; 0.021; 0.022; 0.02 |
| Δ*dotA* | 2 | 0.046  0.047  0.043  0.041 |
| wt | 3 | 0.091  0.094  0.094  0.082 |
| Δ*dotA* | 3 | 0.101  0.107  0.096  0.1 |
| wt | 5 | 0.251  0.266  0.264  0.237 |
| Δ*dotA* | 5 | 0.178  0.181  0.178  0.184 |
| wt | 6 | 0.289  0.308  0.294  0.275 |
| Δ*dotA* | 6 | 0.2  0.203  0.195  0.199 |
| wt | 7 | 0.303  0.327  0.315  0.3 |
| Δ*dotA* | 7 | 0.221  0.213  0.222  0.222 |
| wt | 10 | 0.367  0.369  0.37  0.365 |
| Δ*dotA* | 10 | 0.392  0.386  0.385  0.396 |
| wt | 11 | 0.38  0.38  0.388  0.38 |
| Δ*dotA* | 11 | 0.407  0.389  0.396  0.389 |

**For figure 1B: IL1ß (pg/ml)**

| **Strain** | **Growth phase** | **MOI** | **IL1ß (pg/ml)** |
| --- | --- | --- | --- |
| wt | lag | 3 | 96.96875  88.71875  55.03846  58.96154  13.44491  10.88380  9.94163 |
| wt | lag | 10 | 178.84380  166.71880  117.84620  125.15380  25.26848  24.57844  14.38708 |
| wt | lag | 30 | 245.50000  236.50000  215.73080  195.53850  61.16383  43.52800  47.32322 |
| wt | lag | 100 | 371.59380  384.37500  325.46150  321.23080  82.96644  59.42546  66.45856 |
| wt | log | 3 | 126.96880  118.21880  77.80769  83.57692  38.61810  32.88546  31.00112 |
| wt | log | 10 | 239.71880  243.06250  269.53850  269.34620  75.68121  54.30324  58.73542 |
| wt | log | 30 | 269.50000  275.50000  256.76920  233.23080  133.48530  95.63929  110.67420 |
| wt | log | 100 | 360.12500  345.84380  219.42310  209.80770  131.81330  99.31508  105.52540 |
| wt | Stationary | 3 | 227.25000  236.71880  143.30770  169.88460  46.28816  33.24375  33.70820 |
| wt | stationary | 10 | 248.68750  234.75000  176.26920  170.07690  88.15501  75.50870  68.84716 |
| wt | stationary | 30 | 180.18750  200.15630  140.34620  146.92310  140.66440  116.26090  110.22300 |
| wt | stationary | 100 | 123.71880  119.43750  126.84620  94.80769  83.31146  89.49528  80.79016 |
| Δ*dotA* | lag | 3 | 55.764710  60.205880  17.638890  25.638890  24.123900  17.126350  18.013090 |
| Δ*dotA* | lag | 10 | 147.000000  124.205900  65.972220  74.138890  36.827440  36.499730  32.027470 |
| Δ*dotA* | lag | 30 | 188.176500  169.794100  176.833300  181.000000  72.489890  66.128480  75.863370 |
| Δ*dotA* | lag | 100 | 156.000000  125.911800  308.944500  299.250000  86.562100  82.687420  112.200500 |
| Δ*dotA* | log | 3 | 40.735290  53.147060  40.916670  34.638890  10.013130  9.801088  9.415548 |
| Δ*dotA* | log | 10 | 132.205900  141.823500  111.388900  132.333300  38.292500  55.487580  45.174380 |
| Δ*dotA* | log | 30 | 176.117600  170.176500  195.083300  197.416700  81.550080  89.877750  106.610200 |
| Δ*dotA* | log | 100 | 177.029400  170.529400  249.944400  250.305600  123.381200  148.961700  119.834200 |
| Δ*dotA* | stationary | 3 | 132.823500  112.411800  144.027800  172.250000  43.612950  38.099720  40.798500 |
| Δ*dotA* | stationary | 10 | 198.000000  198.617600  249.722200  246.055600  85.135600  82.378990  96.856020 |
| Δ*dotA* | stationary | 30 | 163.764700  170.088200  217.277800  211.027800  152.450900  131.631700  126.465500 |
| Δ*dotA* | Stationary | 100 | 114.558800  101.735300  195.027800  194.222200  100.518600  96.971680  95.063260 |

**For figure 1 C: IL1ß (pg/ml)**

| **Strain** | **Growth phase** | **IL1ß (pg/ml)** |
| --- | --- | --- |
| wt | lag | 96.96875  88.71875  55.03846  58.96154  13.44491  10.88380  9.94163 |
| wt | log | 126.96880  118.21880  77.80769  83.57692  38.61810  32.88546  31.00112 |
| wt | stationary | 227.25000  236.71880  143.30770  169.88460  46.28816  33.24375  33.70820 |
| Δ*dotA* | lag | 55.764710  60.205880  17.638890  25.638890  24.123900  17.126350  18.013090 |
| Δ*dotA* | log | 40.735290  53.147060  40.916670  34.638890  10.013130  9.801088  9.415548 |
| Δ*dotA* | stationary | 132.823500  112.411800  144.027800  172.250000  43.612950  38.099720  40.798500 |

**For figure 2 A: qPCR data: fold change IL1ß**

| **OMV** | **Δ*flaA*** | **wt** | **Δ*dotA*** | **wt+Δ*flaA*** | **Δ*dotA*+ Δ*flaA*** | **wt+OMV** | **Δ*dotA*+**  **OMV** |
| --- | --- | --- | --- | --- | --- | --- | --- |
| 12432.040 | 22480.550 | 3792.8530 | 4675.6130 | 62561.460 | 61856.670 | 68833.750 | 72486.020 |
| 13912.490 | 24560.340 | 3848.6290 | 7343.1740 | 47839.890 | 59978.670 | 71797.300 | 67351.220 |
| 18596.780 | 28708.710 | 5178.7910 | 2586.7410 | 57304.020 | 41904.570 | 94008.990 | 111872.10 |
| 21419.170 | 22259.480 | 5170.7710 | 2722.6510 | 76843.750 | 59014.250 | 95545.530 | 116093.60 |
| 9158.5740 | 32899.800 | 5780.5970 | 1875.7300 | 50030.400 | 49433.910 | 90179.480 | 56906.010 |
| 9582.1560 | 28622.760 | 5838.1080 | 1703.9190 | 44411.810 | 49322.610 | 103192.40 | 67254.040 |

**For figure 2 B: IL1ß (pg/ml)**

| **mock** | **OMV** | **Δ*flaA*** | **wt** | **Δ*dotA*** | **wt+Δ*flaA*** | **Δ*dotA*+ Δ*flaA*** | **wt+OMV** | **Δ*dotA*+**  **OMV** |
| --- | --- | --- | --- | --- | --- | --- | --- | --- |
| 0 | 37.71429 | 439.8929 | 20.03572 | 47.85714 | 1118.3930 | 1131.9640 | 1793.929 | 2153.750 |
| 0 | 37.46429 | 439.8929 | 20.00000 | 52.85714 | 1054.6430 | 1078.3930 | 1651.964 | 2369.643 |
| 0 | 73.52734 | 306.3242 | 21.96744 | 49.04286 | 848.6898 | 811.9846 | 1237.764 | 1526.871 |
| 0 | 68.77727 | 298.6377 | 30.38802 | 47.92012 | 730.3697 | 899.8610 | 1309.447 | 1614.747 |
| 0 | 27.15676 | 222.6661 | 54.12567 | 31.05532 | 3298.8440 | 2919.6890 | 3044.749 | 2341.325 |
| 0 | 25.18454 | 195.3761 | 57.61145 | 29.75580 | 3143.0540 | 2825.2060 | 3243.041 | 3026.402 |
| 0 | 34.83158 | 189.9334 | 47.90325 | 30.44378 | 3230.1980 | 2689.1380 | 3181.886 | 2953.170 |

**For figure 3 A: WB images provided in SUPPORTING INFORMATION – RAW IMAGES**

**For figure 3 B: Densitometry Data**

|  | **mock** | **OMV** | ***L.p.* Δ*flaA*** | **wt** | **Δ*dotA*** | **wt+**  **Δ*flaA*** | **Δ*dotA*+ Δ*flaA*** | **wt+OMV** | **Δ*dotA*+ OMV** |
| --- | --- | --- | --- | --- | --- | --- | --- | --- | --- |
| **GBP1** | 0.000 | 0.387 | 0.022 | 0.340 | 0.212 | 0.221 | 0.211 | 0.417 | 0.376 |
|  | 0.000 | 0.158 | 0.000 | 0.020 | 0.047 | 0.046 | 0.046 | 0.333 | 0.359 |
| **Casp.-11** | 0.000 | 1.854 | 0.747 | 0.781 | 0.820 | 0.812 | 1.127 | 1.681 | 1.953 |
|  | 0.000 | 2.940 | 0.992 | 1.217 | 1.111 | 1.565 | 1.842 | 2.710 | 2.611 |
|  | 0.000 | 1.899 | 1.132 | 1.314 | 1.267 | 1.340 | 1.520 | 1.910 | 2.671 |
| **Cleaved**  **GSDMD** | 0.000 | 0.385 | 1.151 | 0.002 | 0.061 | 2.971 | 2.391 | 1.617 | 1.606 |
|  | 0.000 | 0.549 | 0.850 | 0.118 | 0.098 | 1.306 | 1.148 | 0.873 | 1.680 |
|  | 0.000 | 0.124 | 0.148 | 0.000 | 0.000 | 0.882 | 1.355 | 1.396 | 1.392 |
|  | 0.000 | 0.194 | 0.182 | 0.000 | 0.000 | 0.729 | 0.940 | 1.211 | 1.109 |
| **NLRP3** | 0.052 | 0.355 | 0.656 | 0.378 | 0.336 | 0.796 | 0.758 | 0.504 | 0.405 |
|  | 0.075 | 0.334 | 0.560 | 0.265 | 0.513 | 1.335 | 0.746 | 0.686 | 0.486 |
| **Uncl.**  **IL1ß** | 0.000 | 1.247 | 1.844 | 0.000 | 0.000 | 0.939 | 1.165 | 1.684 | 1.262 |
|  | 0.000 | 1.591 | 2.262 | 0.012 | 0.058 | 1.376 | 1.346 | 0.984 | 1.095 |
|  | 0.000 | 0.427 | 1.352 | 0.770 | 0.895 | 1.014 | 1.853 | 2.457 | 1.174 |
| **Cleaved**  **IL1ß** | 0.000 | 0.148 | 0.905 | 0.338 | 0.717 | 1.486 | 1.566 | 2.214 | 2.345 |
|  | 0.000 | 0.433 | 0.861 | 0.360 | 0.730 | 1.894 | 1.531 | 1.932 | 2.428 |
|  | 0.000 | 0.394 | 0.776 | 0.061 | 0.237 | 0.674 | 0.999 | 1.517 | 1.340 |

**For figure 4 A: qPCR data: fold change IL1ß**

| OMV | Δ*flaA* | wt | Δ*dotA* | wt+Δ*flaA* | Δ*dotA*+ Δ*flaA* | wt+OMV | Δ*dotA*+  OMV |
| --- | --- | --- | --- | --- | --- | --- | --- |
| 1217.8930 | 2937.2030 | 496.0098 | 744.1749 | 8037.8530 | 18144.3400 | 25715.680 | 37355.880 |
| 1566.1880 | 3584.1830 | 378.7342 | 736.4119 | 13667.5400 | 25508.3200 | 27918.310 | 48224.820 |
| 745.8700 | 1065.7460 | 1621.7420 | 280.0967 | 9734.9730 | 11300.2300 | 24903.750 | 15781.610 |
| 792.1721 | 1123.7200 | 1590.4900 | 225.7690 | 9239.7700 | 7303.0490 | 13952.720 | 16633.920 |
| 1193.9160 | 2484.9900 | 1734.6780 | 551.0623 | 16541.5600 | 30769.3400 | 48235.320 | 33089.070 |
| 1214.9160 | 2173.7190 | 1569.3220 | 447.2871 | 17571.3000 | 28088.7500 | 42892.590 | 37636.700 |

**For figure 4 B: Relative comparison IL1ß mRNA levels H vs N**

| OMV | Δ*flaA* | wt | Δ*dotA* | wt+Δ*flaA* | Δ*dotA*+ Δ*flaA* | wt+OMV | Δ*dotA*+  OMV |
| --- | --- | --- | --- | --- | --- | --- | --- |
| -0.902 | -0.869 | -0.869 | -0.841 | -0.872 | -0.707 | -0.626 | -0.485 |
| -0.887 | -0.854 | -0.902 | -0.900 | -0.714 | -0.575 | -0.611 | -0.284 |
| -0.960 | -0.963 | -0.687 | -0.892 | -0.830 | -0.730 | -0.735 | -0.859 |
| -0.963 | -0.950 | -0.692 | -0.917 | -0.880 | -0.876 | -0.854 | -0.857 |
| -0.870 | -0.924 | -0.700 | -0.706 | -0.669 | -0.378 | -0.465 | -0.419 |
| -0.873 | -0.924 | -0.731 | -0.737 | -0.604 | -0.431 | -0.584 | -0.440 |

**For figure 4 C: IL1ß (pg/ml)**

| Mock | OMV | Δ*flaA* | wt | Δ*dotA* | wt+Δ*flaA* | Δ*dotA*+ Δ*flaA* | wt+OMV | Δ*dotA*+  OMV |
| --- | --- | --- | --- | --- | --- | --- | --- | --- |
| 0. | 0.00000 | 0.0000 | 0.00000 | 0.00000 | 0.0000 | 0.0000 | 34.26786 | 0.00000 |
| 0. | 0.00000 | 0.0000 | 0.00000 | 0.00000 | 0.0000 | 0.0000 | 13.33929 | 0.00000 |
| 0. | 0.00000 | 0.0000 | 0.00000 | 0.00000 | 0.0000 | 0.0000 | 45.08000 | 7.02000 |
| 0. | 0.00000 | 0.0000 | 0.00000 | 0.00000 | 0.0000 | 0.0000 | 25.38000 | 0.76000 |
| 0. | 0.00000 | 0.0000 | 0.00000 | 0.00000 | 0.0000 | 0.0000 | 55.35714 | 84.57143 |
| 0. | 0.00000 | 0.0000 | 0.00000 | 0.00000 | 0.0000 | 0.0000 | 44.60714 | 74.64286 |

**For figure 4 D: Relative comparison IL1ß levels H vs N**

| OMV | Δ*flaA* | wt | Δ*dotA* | wt+Δ*flaA* | Δ*dotA*+ Δ*flaA* | wt+OMV | Δ*dotA*+  OMV |
| --- | --- | --- | --- | --- | --- | --- | --- |
| -1 | -1 | -1 | -1 | -1 | -1 | -0.981 | -1.000 |
| -1 | -1 | -1 | -1 | -1 | -1 | -0.992 | -1.000 |
| -1 | -1 | -1 | -1 | -1 | -1 | -0.964 | -0.995 |
| -1 | -1 | -1 | -1 | -1 | -1 | -0.981 | -1.000 |
| -1 | -1 | -1 | -1 | -1 | -1 | -0.982 | -0.964 |
| -1 | -1 | -1 | -1 | -1 | -1 | -0.986 | -0.975 |

**For figure 5 A:** **WB images provided in SUPPORTING INFORMATION – RAW IMAGES**

**For figure 5 B: Densitometry Data**

|  | **mock** | **OMV** | ***L.p.* Δ*flaA*** | **wt** | **Δ*dotA*** | **wt+Δ*flaA*** | **Δ*dotA*+ Δ*flaA*** | **wt+OMV** | **Δ*dotA*+ OMV** |
| --- | --- | --- | --- | --- | --- | --- | --- | --- | --- |
| **GBP1** | 0.016 | 0.147 | 0.207 | 0.117 | 0.222 | 0.339 | 0.104 | 0.041 | 0.050 |
|  | 0.000 | 0.030 | 0.007 | 0.003 | 0.002 | 0.010 | 0.007 | 0.003 | 0.000 |
|  | 0.000 | 0.378 | 0.000 | 0.000 | 0.000 | 0.008 | 0.000 | 0.318 | 0.269 |
| **Casp.-11** | 0.000 | 1.410 | 0.781 | 0.902 | 1.965 | 1.815 | 1.942 | 2.084 | 2.369 |
|  | 0.000 | 2.091 | 1.299 | 1.631 | 1.686 | 1.417 | 2.335 | 2.571 | 2.061 |
| **Cleaved**  **GSDMD** | 0.000 | 0.000 | 0.278 | 0.000 | 0.000 | 0.000 | 0.000 | 0.221 | 0.181 |
|  | 0.000 | 0.000 | 0.000 | 0.000 | 0.000 | 0.000 | 0.000 | 1.070 | 0.243 |
|  | 0.000 | 0.067 | 0.152 | 0.000 | 0.000 | 0.000 | 0.000 | 1.216 | 0.770 |
| **NLRP3** | 0.041 | 0.260 | 0.152 | 0.087 | 0.548 | 1.774 | 0.421 | 0.571 | 0.694 |
|  | 0.059 | 0.488 | 0.570 | 0.690 | 1.108 | 2.136 | 2.645 | 1.652 | 1.311 |
|  | 0.136 | 0.558 | 0.579 | 0.624 | 1.183 |  | 1.954 | 1.047 | 0.647 |
| **Uncl.**  **IL1ß** | 0.000 | 0.706 | 0.338 | 0.755 | 0.413 | 0.609 | 0.809 | 5.705 | 2.501 |
|  | 0.000 | 0.987 | 1.546 | 1.156 | 2.377 |  | 3.572 | 1.886 | 1.466 |
|  | 0.000 | 0.511 | 0.593 | 0.998 | 0.591 | 6.938 | 6.696 | 5.682 | 3.244 |
| **Cleaved**  **IL1ß** | 0.000 | 0.000 | 0.000 | 0.000 | 0.000 | 0.000 | 0.000 | 0.993 | 0.903 |
|  | 0.000 | 0.000 | 0.000 | 0.000 | 0.000 | 0.646 | 0.476 | 2.202 | 4.443 |
|  | 0.000 | 0.000 | 0.000 | 0.000 | 0.000 | 0.000 | 0.000 | 0.000 | 0.000 |

**For figure 6: qPCR data: fold change**

|  | **OMV** | **wt** | **wt+OMV** | **Δ*dotA*** | **Δ*dotA*+OMV** |
| --- | --- | --- | --- | --- | --- |
| *Gate-16* | 1.515893 | 1.354199 | 1.938797 | 1.318533 | 1.066486 |
|  | 1.493060 | 1.589044 | 2.051007 | 1.514394 | 1.252976 |
|  | 1.265301 | 1.548252 | 1.771912 | 1.573246 | 2.932489 |
|  | 1.541934 | 1.687022 | 3.293618 | 1.845905 | 1.393633 |
|  | 1.228392 | 1.616301 | 1.559623 | 1.517794 | 1.498463 |
|  | 1.304250 | 1.484662 | 1.642627 | 1.356644 |  |
| *Irgm2* | 27.720570 | 6.439364 | 27.867650 | 4.370016 | 20.130570 |
|  | 28.756700 | 6.242579 | 29.167760 | 5.462557 | 22.692360 |
|  | 24.058850 | 5.449416 | 25.275460 | 10.514710 | 44.539080 |
|  | 25.375890 | 6.701113 | 49.672430 | 9.119628 | 26.916250 |
|  | 31.284810 | 5.475508 | 22.430510 | 6.944452 | 27.202840 |
|  | 24.764400 | 3.855381 | 19.677770 | 6.217654 |  |
| *Serpinb1* | 0.6865047 | 0.03889056 | 0.7303894 | 0.0383133 | 0.5505499 |
|  | 0.8747368 | 0.05041291 | 0.7029391 | 0.03832732 | 0.7374726 |
|  | 1.300324 | 0.05675828 | 2.214258 | 0.09623228 | 1.333580 |
|  | 1.088144 | 0.04250231 | 2.408135 | 0.06701054 | 0.2491403 |
|  | 1.011243 | 0.04870749 | 1.201397 | 0.02919002 | 0.2610725 |
|  | 1.241430 | 0.04087144 | 1.049635 | 0.04821533 |  |
| *TRIM21* | 10.879320 | 1.769860 | 11.374350 | 1.321347 | 15.047570 |
|  | 14.080210 | 2.247408 | 14.777930 | 1.968992 | 21.391110 |
|  | 12.640780 | 2.257733 | 12.843340 | 3.384020 | 28.387500 |
|  | 11.971130 | 3.099858 | 24.126080 | 4.596511 | 22.118640 |
|  | 17.692050 | 2.135367 | 11.453250 | 2.562053 | 21.336750 |
|  | 13.514720 | 1.819492 | 9.975892 | 2.051636 |  |

**For figure 7 A: IL1ß (pg/ml)**

|  | mock | OMV | wt | wt+OMV | Δ*dotA* | Δ*dotA*+OMV | LPS+Nig. |
| --- | --- | --- | --- | --- | --- | --- | --- |
| control | 0.000000 | 57.389310 | 495.399100 | 3065.408000 | 137.039700 | 3335.891000 | 13046.51000 |
|  | 0.000000 | 56.374310 | 469.815700 | 3396.754000 | 135.162500 | 3125.859000 | 14758.75000 |
|  | 0.000000 | 17.025270 | 50.136320 | 1818.947000 | 125.969100 | 2165.609000 | 14374.75000 |
|  | 0.000000 | 6.612866 | 59.109530 | 1859.612000 | 134.836100 | 2079.161000 | 15657.04000 |
|  | 0.000000 | 16.841750 | 51.411310 | 1656.386000 | 161.234100 | 2004.304000 |  |
|  | 0.000000 | 9.829313 | 58.761810 | 1676.187000 | 139.984300 | 1747.857000 |  |
| ACOD1^-/-^ | 0.000000 | 26.862850 | 213.765100 | 1819.663000 | 193.825200 | 1987.812000 | 12340.0200 |
|  | 0.000000 | 28.314410 | 199.282200 | 1876.401000 | 168.297400 | 2258.088000 | 12269.4900 |
|  | 0.000000 | 9.790677 | 1.619163 | 255.348500 | 183.276000 | 1768.334000 | 12297.4100 |
|  | 0.000000 | 7.163429 | 4.526522 | 232.166900 | 186.424800 | 1729.312000 | 12753.9000 |
|  | 0.000000 | 23.013110 | 7.769940 | 265.011300 | 190.563000 | 1370.974000 |  |
|  | 0.000000 | 17.890400 | 6.458028 | 234.087600 | 180.630000 | 1446.149000 |  |
|  | 0.000000 | 35.278440 | 21.357600 | 241.167800 | 250.494500 | 1074.753000 |  |
|  | 0.000000 | 34.029000 | 28.146220 | 243.458400 | 268.923700 | 1091.308000 |  |

**For figure 7 B: WB images provided in SUPPORTING INFORMATION – RAW IMAGES**

**For figure 7 B: densitometry data:**

|  | **Mock** | **OMV** | **wt** | **Δ*dotA*** | **wt+OMV** | **Δ*dotA***  **+OMV** | **LPS+Nig** |
| --- | --- | --- | --- | --- | --- | --- | --- |
| **ctrl**  **Caspase-11** | 0.00 | 1.55 | 0.33 | 0.52 | 2.08 | 1.37 | 0.03 |
|  | 0.00 | 0.698 | 0.004 | 0.007 | 0.341 | 0.240 | 0.000 |
|  | 0.00 | 1.28 | 0.25 | 0.46 | 1.56 | 1.13 | 0.72 |
| **Ctrl cl. GSDMD** | 0.00 | 0.00 | 0.00 | 0.00 | 0.06 | 0.05 | 1.05 |
|  | 0.00 | 0.00 | 0.000 | 0.000 | 0.165 | 0.030 | 1.880 |
|  | 0.000 | 0.013 | 0.063 | 0.100 | 0.554 | 0.682 | 1.113 |
| **ACOD1^-/-^**  **Caspase-11** | 0.000 | 1.397 | 0.464 | 0.421 | 1.824 | 0.924 | 0.362 |
|  | 0.000 | 1.365 | 0.223 | 0.036 | 1.282 | 0.712 | 0.026 |
|  | 0.000 | 0.641 | 0.289 | 0.048 | 1.134 | 0.442 | 0.000 |
| **ACOD1^-/-^**  **cl. GSMD** | 0.000 | 0.003 | 0.012 | 0.010 | 0.100 | 0.085 | 1.329 |
|  | 0.000 | 0.000 | 0.000 | 0.000 | 0.031 | 0.020 | 0.681 |
|  | 0.000 | 0.000 | 0.015 | 0.014 | 0.115 | 0.100 | 0.765 |

**For figure 7 C: CFU**

|  | **wt** | **wt+OMV** | **Δ*dotA*** | **Δ*dotA*+OMV** |
| --- | --- | --- | --- | --- |
| Control 32h | 873333.3000 | 815000.000 | 205000. | 220000.0000 |
|  | 660000.0000 | 930000.000 | 280000. | 180000.0000 |
|  | 650000.0000 | 1010000.000 | 160000. | 190000.0000 |
|  | 595000.0000 | 405000.000 | 160000. | 280000.0000 |
|  | 490000.0000 | 440000.000 | 125000. | 225000.0000 |
|  | 720000.0000 |  | 130000. |  |
|  | 645000.0000 | 515000.000 | 240000. | 173333.3000 |
|  | 740000.0000 | 490000.000 | 130000. | 205000.0000 |
|  | 670000.0000 |  | 175000. |  |
| Control 96h | 480000.00 | 245000.0000 | 233500.00000 | 237333.3000 |
|  | 430000.00 | 296500.0000 | 214000.00000 | 148000.0000 |
|  | 256000.00 | 317333.3000 | 209000.00000 | 138500.0000 |
|  | 415000.00 | 34000.0000 | 53333.33000 | 35000.0000 |
|  | 265000.00 | 47500.0000 | 71500.00000 | 43500.0000 |
|  | 290000.00 |  | 88500.00000 |  |
|  | 215000.00 | 126666.7000 | 90000.00000 | 18000.0000 |
|  | 275000.00 | 80000.0000 | 97000.00000 | 23000.0000 |
|  | 265000.00 | 21500.0000 | 153333.30000 | 19500.0000 |
| ACOD1^-/-^ 32h | 2866667.000 | 3800000.000 | 1100000. | 800000. |
|  | 2950000.000 | 2750000.000 | 1500000. | 830000. |
|  | 3450000.000 |  | 1000000. |  |
|  | 3150000.000 | 2750000.000 | 850000. | 795000. |
|  | 2800000.000 | 2700000.000 | 740000. | 895000. |
|  | 2733333.000 |  | 600000. |  |
|  | 3100000.000 | 4250000.000 | 1550000. | 1050000. |
|  | 3600000.000 | 2300000.000 | 1250000. | 1400000. |
|  | 3100000.000 |  | 1400000. |  |
| ACOD1^-/-^ 96h | 2.200000e+007 | 1050000.0000 | 655000.0000 | 250000.0000 |
|  | 2.650000e+007 | 666666.7000 | 580000.0000 | 480000.0000 |
|  | 3.250000e+007 | 700000.0000 | 666666.7000 | 315000.0000 |
|  | 2.550000e+007 | 900000.0000 | 680000.0000 | 285000.0000 |
|  | 2.850000e+007 | 505000.0000 | 1150000.0000 | 375000.0000 |
|  | 4.000000e+007 | 800000.0000 | 1000000.0000 | 425000.0000 |
|  | 4.666667e+007 | 370000.0000 | 420000.0000 | 390000.0000 |
|  | 4.850000e+007 | 475000.0000 | 510000.0000 | 280000.0000 |
|  | 2.050000e+007 | 575000.0000 | 810000.0000 | 253333.3000 |

**For figure 8 A: Cell viability (OD_550_)**

| wt 24h | wt-OMV 48h | wt+OMV 48h | wt-OMV 96h | wt+OMV 96h |
| --- | --- | --- | --- | --- |
| 0.17785 | 0.16210 | 0.12100 | 0.17350 | 0.10670 |
| 0.16650 | 0.15590 | 0.12905 | 0.17785 | 0.12015 |
| 0.17975 | 0.16215 | 0.11960 | 0.17195 | 0.11360 |
| 0.15985 | 0.18990 |  | 0.19790 | 0.13415 |
| 0.16380 |  |  |  |  |
| 0.16830 |  |  |  |  |
| 0.20210 | 0.16815 | 0.12710 | 0.22505 | 0.13135 |
| 0.18125 | 0.16315 | 0.15545 | 0.19770 | 0.12330 |
| 0.17620 | 0.16445 | 0.15030 | 0.21215 | 0.12860 |
| 0.17680 | 0.17445 |  | 0.21585 | 0.12035 |
| 0.18605 |  |  |  |  |
| 0.17840 |  |  |  |  |
| 0.18945 |  |  |  |  |
| 0.20395 |  |  |  |  |
| 0.26090 | 0.31995 | 0.29105 | 0.43430 | 0.17625 |
| 0.27695 | 0.37160 | 0.28040 | 0.38020 | 0.14790 |
| 0.23830 | 0.37885 | 0.28790 | 0.39760 | 0.13275 |
| 0.27835 | 0.37930 | 0.30325 | 0.42110 | 0.14440 |
| 0.39745 | 0.31940 | 0.25730 | 0.50155 | 0.11730 |
| 0.27750 | 0.35170 | 0.28595 | 0.42605 | 0.12275 |
| 0.27755 | 0.33635 | 0.28805 | 0.47080 | 0.12380 |
| 0.30900 | 0.36910 | 0.26325 | 0.47855 | 0.10330 |

**For figure 8 B: LDH release (OD_400_**)

| wt 24h | wt-OMV 48h | wt+OMV 48h | wt-OMV 96h | wt+OMV 96h |
| --- | --- | --- | --- | --- |
| 0.15109170 | 0.3267500 | 0.4761000 | 0.4172500 | 0.6564250 |
| 0.17436250 | 0.3541375 | 0.4947375 | 0.4585625 | 0.8014875 |
| 0.27371250 | 0.2403250 | 0.4454875 | 0.5796250 | 1.1987120 |
| 0.21908750 | 0.3165250 | 0.5035125 | 0.3893250 | 1.4947380 |

**For figure 9 A: Relative CFU (%)**

| **48h: wt** | **48h: wt + OMV** | **96h: wt** | **96h: wt + OMV** |
| --- | --- | --- | --- |
| 96.06 | 91.63 | 96.06 | 37.44 |
| 113.79 | 50.25 | 69.46 | 39.90 |
| 90.15 | 56.16 | 97.54 | 38.42 |
|  |  | 81.28 | 39.41 |
| 100.56 | 72.05 | 114.82 | 66.79 |
| 111.82 | 66.04 | 87.05 | 26.27 |
| 96.81 | 52.53 | 55.53 | 24.77 |
| 90.81 |  | 131.33 | 28.52 |
| 107.19 | 62.75 | 70.59 | 18.82 |
| 96.73 | 65.36 | 57.52 | 34.25 |
| 96.73 | 60.13 | 59.26 | 29.02 |
| 99.35 | 60.13 | 60.13 | 39.22 |
| 91.67 | 83.33 | 58.33 | 16.83 |
| 71.67 | 66.67 | 58.33 | 12.50 |
| 118.33 | 76.67 | 63.33 | 13.17 |
| 118.33 | 90.00 | 60.00 | 15.00 |

**For figure 9 B: Relative CFU (%)**

| **cells: wt** | **cells: wt + OMV** | **SN: wt** | **SN: wt + OMV** |
| --- | --- | --- | --- |
| 81.44 | 25.63 | 55.09 | 26.35 |
| 117.37 | 23.95 | 47.43 | 30.66 |
| 100.60 | 35.93 | 36.41 | 28.26 |
| 100.60 | 26.59 | 46.95 | 22.51 |
| 111.39 | 44.55 | 92.08 | 41.58 |
| 90.59 | 38.61 | 109.90 | 23.17 |
| 98.02 | 50.50 | 56.44 | 17.82 |
|  | 26.73 | 65.35 | 17.23 |
| 90.48 | 15.56 | 73.02 | 15.56 |
| 104.76 | 52.38 | 60.32 | 18.73 |
| 101.59 | 34.92 | 30.16 | 22.86 |
| 103.17 | 39.68 | 85.71 | 20.32 |

**For figure 10 A: WB images provided in SUPPORTING INFORMATION – RAW IMAGES**

**For figure 10 B: IL1ß (pg/ml)**

| **mock** | **OMV** | **wt** | **Mut1** | **Mut2** |
| --- | --- | --- | --- | --- |
| 0.00 | 111.82 | 16.36 | 31.21 |  |
| 0.00 | 93.94 | 11.21 | 33.03 | 121.82 |
| 0.00 | 74.55 | 9.39 | 40.91 | 29.39 |
| 0.00 |  |  | 32.42 | 32.12 |
| 0.00 | 97.05 | 14.21 | 40.34 | 33.75 |
| 0.00 | 89.66 | 17.39 | 35.80 | 39.32 |
| 0.00 | 144.21 | 90.53 | 173.95 | 146.84 |
| 0.00 | 138.95 | 94.47 | 163.95 | 189.47 |
| 0.00 | 159.21 | 97.90 | 190.26 | 193.95 |
| 0.00 | 165.53 | 56.84 | 56.58 | 58.68 |
| 0.00 | 113.16 | 75.53 | 58.95 | 64.21 |
| 0.00 | 171.58 | 73.95 | 62.90 | 67.11 |
| 0.00 | 178.65 | 30.39 | 83.08 | 84.62 |
| 0.00 | 206.35 | 30.77 | 85.38 | 112.31 |
| 0.00 | 222.50 | 33.85 | 84.42 | 105.39 |
| 0.00 | 107.20 | 48.60 | 80.00 | 85.20 |
| 0.00 | 118.20 | 33.00 | 85.80 | 84.60 |
| 0.00 | 114.00 | 41.80 | 84.40 | 86.80 |

**For figure 10 C: CFU**

| **wt** | **wt + OMV** | **Mut1** | **Mut1 + OMV** | **Mut2** | **Mut2 + OMV** |
| --- | --- | --- | --- | --- | --- |
| 200000 | 200000 | 61300 | 147000 | 60000 | 160000 |
| 160000 | 227000 | 84000 | 160000 | 66700 | 213000 |
| 133000 | 400000 | 86700 | 120000 | 65300 | 187000 |
| 360000 | 80000 | 133000 | 133000 | 160000 | 213000 |
| 427000 | 86700 | 54700 | 173000 | 107000 | 240000 |
| 400000 | 213000 | 160000 | 160000 | 80000 | 147000 |
| 360000 | 80000 | 93300 | 80000 | 80000 | 34700 |
| 253000 | 28000 | 120000 | 66700 | 107000 | 58700 |
| 373000 | 107000 | 187000 | 80000 | 147000 | 52000 |
| 160000 | 25300 | 107000 | 21300 | 80000 | 25300 |
| 333000 | 34700 | 187000 | 42700 | 93300 | 33300 |
| 107000 | 18700 | 147000 | 26700 | 80000 | 25300 |

**For figure 10 D: IL1ß ELISA (pg/ml)**

|  | **wt** | **Mut1** | **Mut1 induced** | **Mut2** | **Mut2 induced** |
| --- | --- | --- | --- | --- | --- |
| **24 h** | 8.23 | 6.80 | 16.94 | 5.94 | 23.23 |
|  | 7.80 | 5.08 | 17.51 | 6.51 | 23.37 |
|  | 8.60 | 3.60 | 22.39 | 4.36 | 14.89 |
|  | 2.76 | 2.99 | 11.78 | 4.81 | 12.61 |
| **48 h** | 6.08 | 5.80 | 37.80 | 7.94 | 40.94 |
|  | 6.08 | 7.65 | 38.23 | 11.23 | 46.08 |
|  | 6.40 | 5.42 | 18.80 | 7.54 | 24.28 |
|  | 4.13 | 4.74 | 19.74 | 6.78 | 22.92 |
| **72 h** | 8.51 | 7.80 | 43.23 | 11.08 | 36.80 |
|  | 5.80 | 7.80 | 45.80 | 21.33 | 43.80 |
|  | 4.51 | 6.02 | 24.36 | 7.54 | 24.74 |
|  | 5.42 | 5.11 | 20.42 | 8.52 | 20.19 |
| **96 h** | 11.08 | 7.65 | 42.65 | 7.80 | 33.37 |
|  | 7.23 | 3.37 | 30.94 | 12.65 | 34.94 |
|  | 4.43 | 5.04 | 17.16 | 6.86 | 6.10 |
|  | 6.02 | 6.02 | 17.54 | 6.78 | 16.10 |

**For figure 10 E: CFU**

|  | **wt** | **Mut1** | **Mut1 induced** | **Mut2** | **Mut2 induced** |
| --- | --- | --- | --- | --- | --- |
| **24 h** | 30000000 | 40000000 | 50000000 | 50000000 | 10000000 |
|  | 50000000 | 50000000 | 40000000 | 30000000 | 9000000 |
|  | 10000000 | 10000000 | 10000000 | 6000000 | 4000000 |
|  | 10000000 | 8000000 | 10000000 | 7000000 | 800000 |
| **96 h** | 500000 | 700000 | 200000 | 900000 | 40000 |
|  | 500000 | 900000 | 200000 | 500000 | 30000 |
|  | 300000 | 100000 | 50000 | 500000 | 100000 |
|  | 300000 | 700000 | 200000 | 1000000 | 100000 |
